# Single-cell transcriptomic profiling reveals the tumor heterogeneity of small-cell lung cancer

**DOI:** 10.1038/s41392-022-01150-4

**Published:** 2022-10-05

**Authors:** Yanhua Tian, Qingqing Li, Zhenlin Yang, Shu Zhang, Jiachen Xu, Zhijie Wang, Hua Bai, Jianchun Duan, Bo Zheng, Wen Li, Yueli Cui, Xin Wang, Rui Wan, Kailun Fei, Jia Zhong, Shugeng Gao, Jie He, Carl M. Gay, Jianjun Zhang, Jie Wang, Fuchou Tang

**Affiliations:** 1 State Key Laboratory of Molecular Oncology, Department of Medical Oncology, National Cancer Center/National Clinical Research Center for Cancer/Cancer Hospital, Chinese Academy of Medical Sciences and Peking Union Medical College, Beijing, China; 2 Biomedical Pioneering Innovation Center, School of Life Sciences, Peking University, Beijing, China; 3 Beijing Advanced Innovation Center for Genomics & Ministry of Education Key Laboratory of Cell Proliferation and Differentiation, Beijing, China; 4 Department of Throacic Surgery, National Cancer Center/National Clinical Research Center for Cancer/Cancer Hospital, Chinese Academy of Medical Sciences and Peking Union Medical College, Beijing, China; 5 Academy for Advanced Interdisciplinary Studies, Peking University, Beijing, China; 6 Department of Thoracic/Head & Neck Medical Oncology, UT MD Anderson Cancer Center, Houston, TX 77030 USA; 7 Peking-Tsinghua Center for Life Sciences, Peking University, Beijing, China

**Keywords:** Lung cancer, Cancer microenvironment

## Abstract

Small-cell lung cancer (SCLC) is the most aggressive and lethal subtype of lung cancer, for which, better understandings of its biology are urgently needed. Single-cell sequencing technologies provide an opportunity to profile individual cells within the tumor microenvironment (TME) and investigate their roles in tumorigenic processes. Here, we performed high-precision single-cell transcriptomic analysis of ~5000 individual cells from primary tumors (PTs) and matched normal adjacent tissues (NATs) from 11 SCLC patients, including one patient with both PT and relapsed tumor (RT). The comparison revealed an immunosuppressive landscape of human SCLC. Malignant cells in SCLC tumors exhibited diverse states mainly related to the cell cycle, immune, and hypoxic properties. Our data also revealed the intratumor heterogeneity (ITH) of key transcription factors (TFs) in SCLC and related gene expression patterns and functions. The non-neuroendocrine (non-NE) tumors were correlated with increased inflammatory gene signatures and immune cell infiltrates in SCLC, which contributed to better responses to immune checkpoint inhibitors. These findings indicate a significant heterogeneity of human SCLC, and intensive crosstalk between cancer cells and the TME at single-cell resolution, and thus, set the stage for a better understanding of the biology of SCLC as well as for developing new therapeutics for SCLC.

## Introduction

Small-cell lung cancer (SCLC) is an extremely aggressive malignant tumor type characterized by rapid growth, the early development of widespread metastases and acquired therapeutic resistance to radiotherapy and chemotherapy.^1^ It has been designated a recalcitrant malignancy due to its poor prognosis and minimal improvement in treatment over the past several decades.^2^ Recently, initial milestones have been achieved in SCLC treatment by incorporating immune checkpoint inhibitors into chemotherapy.^3–5^ However, the clinical benefits are limited, with a mild improvement in overall survival, and only a small number of patients benefit from these immune-based therapies. Currently, there are no reliable biomarkers, such as tumor mutation burden (TMB), and programmed cell death-ligand 1 (*PD-L1*) expression, that can accurately predict clinical outcomes.^6,7^ The recent molecular understanding of SCLC has been translated into only modest clinical improvements, highlighting the urgent need for a better understanding of this recalcitrant malignancy.

While SCLC has been regarded as a genetically homogeneous malignancy with nearly universal loss of *TP53* and *RB1,*^8^ recent transcriptomic profiling studies have suggested the classification of molecular subtypes based on the relative expression of *ASCL1*, *NEUROD1*, *POU2F3*, *YAP1*, and other key transcription factors (TFs).^9–11^ In addition, these subtypes have been shown to mediate distinct vulnerabilities and therapeutic targets, thus promoting the development of subtype-specific drug screens as well as clinical therapeutic studies.^12,13^ Despite substantial achievements, these studies mostly focused on the intertumor heterogeneity of malignant cells at bulk levels, limiting the exploration of intratumor heterogeneity (ITH) and interactions between distinct cell components in the SCLC tumor microenvironment (TME). Substantial evidence indicates that ITH between malignant and nonmalignant cells, and their interactions within the TME are critical to diverse aspects of tumor biology and therapeutic responses.^14^

Single-cell RNA sequencing (scRNA-seq) technologies provide an opportunity to profile cell components within the TME and investigate what roles they play in tumor occurrence and development.^15^ In contrast to standard bulk population sequencing, which provides only average values, scRNA-seq allows the molecular distinction of all cell types within a complex population, including malignant cells, immune cells, and stromal cells in the TME.^16^ In addition, the ITH of these cell types as well as interactions between these multicellular components could also be further investigated by scRNA-seq.^17^ However, while scDNA-seq and scATAC-seq approaches can be applied to archival specimens, most scRNA-seq methods require viable cell suspensions from fresh tissues,^15^ and have been hindered by the lack of surgical specimens of SCLC. While single-cell transcriptomic profiling analyses have been performed on most cancer types, there are still no scRNA-seq studies on matched primary tumors (PTs), normal lung tissues adjacent to the tumor (NATs), and relapsed tumor (RTs) for human SCLC.^18–20^ Here, by performing modified single-cell tagged reverse transcription sequencing (STRT-seq) of fresh samples from primary and matched adjacent normal lung tissues from 11 patients with limited-stage SCLC, we revealed a heterogeneous cellular architecture of this malignant cancer at single-cell resolution.

## Results

### Single-cell gene expression atlas of SCLC

**Fig. 1:**
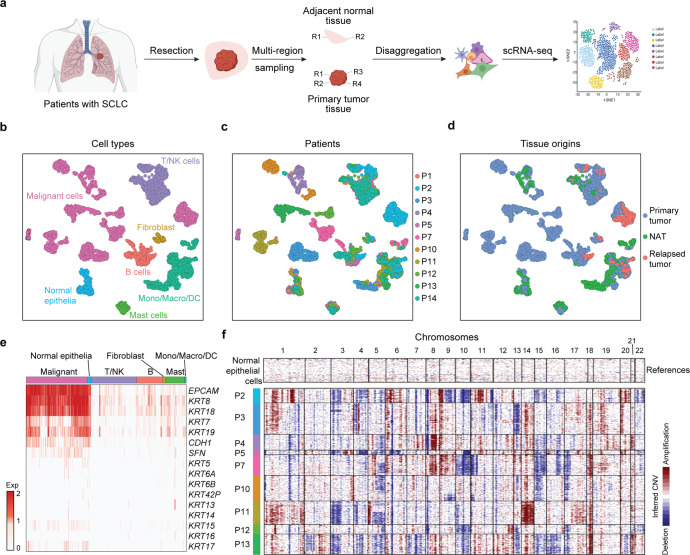
scRNA-seq profiling of the landscape of SCLC. **a** Experimental design. Schematic of the experimental workflow for the collection and processing of fresh tissue samples of SCLC tumors and matched normal lung tissues adjacent to the tumor for scRNA-seq (created with BioRender. com). **b**–**d** UMAP plot of all cells clustered and color coded by cell type (**b**), patient (**b**), and tissue origin (**d**). Clusters were assigned to the indicated cell types by differentially expressed genes (DEGs), as shown in (**b**) (Supplementary Fig. S1b). **e** Epithelial gene expression across all cell types in the TME, including malignant cells (malignant), normal epithelial cells (normal epithelia), T/NK cells (T/NK), B cells (B), fibroblasts, mast cells (Mast), and monocytes/macrophages/dendritic cells (mono/macro/DC). **f** Heatmap shows large-scale CNVs in malignant cells (rows) from 9 of the 11 SCLC patients. There were almost no malignant cells from SCLC-P1 and SCLC-P14 because these two patients received neoadjuvant treatment. The inferred CNVs were deduced for each single cell based on the average expression profiles across chromosomal intervals. Normal epithelia from NATs were used as references. Red: amplification; blue: deletion

To describe the gene expression atlas of SCLC tumors at the single-cell level, we performed modified STRT-seq on matched PTs, NATs and RTs from 11 SCLC patients who underwent surgical resection (Fig. 1a and Supplementary Table S1). All the patients included were males, and most of them (9/11) had a heavy smoking history, consistent with typical SCLC characteristics.^21^ Single-cell transcriptomes from a total of 4911 cells from PTs (*n* = 3365), NATs (*n* = 1274) and relapsed tumors (RTs, *n* = 272) were obtained after initial quality controls (Fig. 1b–d and Supplementary Fig. S1b). In PTs and RTs, we distinguished 1954 malignant and 1683 nonmalignant cells by their epithelial origins, clustering patterns, and inferred large-scale chromosomal copy number variations (CNVs). Epithelial biomarkers, such as *EPCAM* and *KRT8/18*, were uniformly expressed at high levels across all SCLC malignant cells, while *KRT7/19*, *CDH1* and *SFN* were partially expressed in individual malignant cells (Fig. 1e). Other KRT family genes, including *KRT 5/6A/6B/42P/13/14/15/16/17*, which are widely expressed in other solid tumors, such as non-small cell lung cancer (NSCLC) and head and neck squamous cell carcinoma (HNSCC),^22,23^ were rarely expressed in SCLC, representing a cancer type-specific expression profile (Fig. 1e). Notably, each of the malignant clusters contained cells from patient-specific subpopulations, representing a significant intertumor heterogeneity (Fig. 1b, c). In contrast, nonmalignant cells in the TME and cells from the NAT, identified by the expression of known markers, tended to cluster together by cell type, and the same cluster contained cells from different patients, indicating that these cell types and expression states are largely consistent across patients, although they do vary in their proportions (Fig. 1c, d and Supplementary Fig. S1b, c). The inferred CNVs not only confirmed the separation of malignant cells from nonmalignant cells with normal karyotypes but also revealed the widespread heterogeneity of SCLC at the genome level (Fig. 1f and Supplementary Fig. S1d–f). Specifically, while most CNVs showed intertumor heterogeneity, 5q loss exhibited both intertumor heterogeneity and ITH (P3 normal; P2, P4, P5, P12, and P13 with 5q loss, and P7, P10, and P11 with partial 5q loss). We next performed bulk DNA sequencing on multi-region tissues matched to those used for scRNA-seq. The CNVs called from low-pass whole-genome sequencing (WGS) data, including uniform chromosome 10, 11p, 15, 16q, and 17p loss and 8, 9 and 18 gain in P7, 15 and 17p loss in P10, and 1, 14, 17q, and 18 gain in P11 (Supplementary Fig. S1d–f), firmly validated the inferred CNVs from scRNA-seq data. These results also revealed 5q loss, which was associated with an increased risk of recurrence in our cohort, as spatial ITH in SCLC (Supplementary Fig. S1g).

### The immune landscape of SCLC

**Fig. 2:**
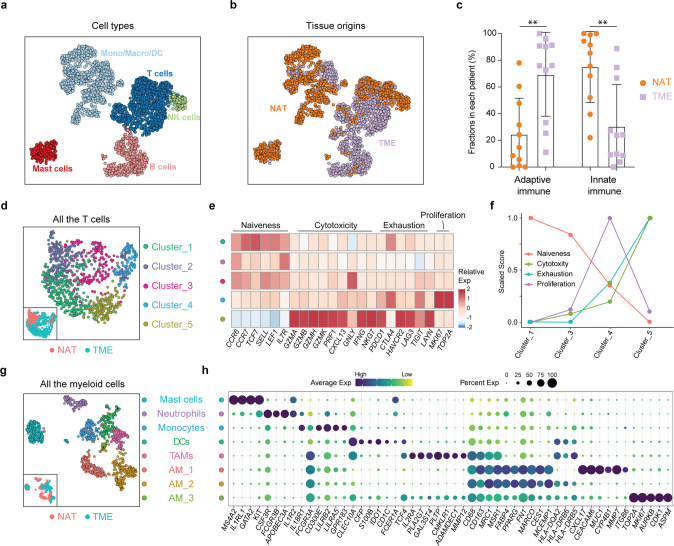
The immune landscape of SCLC. **a**, **b** UMAP plot of 2,474 immune cells colored coded by cell type (T cells, NK cells, mast cells, and mono/macro/DC cells) (**a**) and tissue origin (NAT and TME) (**b**). **c** The fractions of T cells and B/plasma cells were higher and the infiltration of myeloid cells was lower in primary SCLC tumors than in NATs. Statistical analyses were performed using two-way ANOVA followed by Bonferroni’s multiple comparisons test. **p < 0.01. **d** UMAP plot of all T cells from NATs and the TME. **e** Heatmap of the z-score-normalized mean expression value of T cell state-associated genes in each subcluster. The T cell subclusters were colored in accordance with those in (**d)**. **f** The naïveness, cytotoxicity, exhaustion and proliferation scores in T cell subclusters from the TME. Every score was scaled to a range of 0 to 1. **g** UMAP plot of all myeloid cells from NATs and the TME. **h** Dot plot of selected myeloid cell-associated genes in each cell lineage. Dot size and color indicate the fraction of expressing cells and normalized expression levels, respectively

Single-cell profiles of nonmalignant cells highlighted the composition of SCLC (Fig. 2a, b). Compared with NATs, PTs exhibited an increased fraction of lymphocytes and less myeloid cell infiltration (Fig. 2c), indicating a more significant role played by adaptive immunity within the TME and a more significant role played by the innate immune response in normal lung tissues. We then re-clustered the T cells and myeloid cells powered by their relatively large numbers in our dataset. T cells from both NATs and the TME were mainly composed of *CD8*-positive T cells and expressed high levels of cytotoxic markers, such as *GZMA/B/H/K*, *PRF1*, *NKG7*, *IFNG*, *GNLY* and *CXCL13*, implying significant immune surveillance in SCLC (Supplementary Fig. S2a). Compared with only cytotoxic T cells from NATs, we observed the increased diversity of T cells from the TME, including four heterogeneous activation states based on naïveness, cytotoxicity, exhaustion and proliferation properties (Fig. 2d, e). We then calculated the scores of these T cell states based on the average expression levels of marker genes that have been well defined either in our dataset or in previously published studies.^24^ The exhaustion score exhibited a positive correlation with the cytotoxicity score, consistent with observations in metastatic melanoma,^25^ and a negative correlation with the naïveness score (Fig. 2f). Proliferating T cells were characterized by high expression of cell cycle-related genes, such as *MKI67* and *TOP2A*, implying a T cell status that is undergoing extensive clonal expansions. This cell population also expressed high levels of cytotoxic genes and median levels of coinhibitory receptors (Fig. 2e). Next, we examined the expression preference in individual patients. As shown in Supplementary Fig. S2b, the proliferating T cell cluster was composed of T cells from almost all patients included, implying a uniform existence of this T cell status in SCLC patients (Supplementary Fig. S2b). In contrast, other T cell clusters were mainly composed of cells from only one or two patients, for example, cluster 5 was mainly from P2, cluster 1 was from P1 and P14, and cluster 3 was mainly from P7 and P12 (Supplementary Fig. S2b). The detailed classification of T cells in SCLC also revealed the expression patterns of markers of dysfunction and exhaustion (e.g., *PDCD1*, *CTLA4*, *HAVCR2*, *LAG3*, *TIGIT*, and *LAYN*), which might serve as immunotherapy targets (Supplementary Fig. S2c). *HAVCR2* exhibited the highest expression level in the exhausted T cell subcluster (mainly from P2), while *CTLA4* was preferentially expressed in other T cell subclusters from the SCLC TME. *LAYN*, which is associated with the suppressive function of exhausted *CD8* T cells in NSCLC^24^ and liver cancer,^26^ however, exhibited a sporadic expression in SCLC. These heterogeneous states and coinhibitory receptor expression preferences of SCLC T cells not only provide potential immunotherapy targets for SCLC, but also suggest that there might be increased benefits for selected patients who respond to immunotherapy.

We next performed unsupervised clustering analysis of myeloid cells from both NATs and the TME and identified more heterogeneous cell types based on well-annotated marker genes.^17^ Re-clustering revealed essentially all major known myeloid cell lineages, including mast cells, neutrophils, monocytes, dendritic cells (DCs), and macrophages. Further clustering revealed four distinct macrophage clusters: three lung-resident alveolar macrophages (AM_1, AM_2, and AM_3) and one tumor-associated macrophage (TAM) (Fig. 2g, Supplementary Fig. S2d, e). By using the single-cell data from a published study,^18^ we also validated the existence of three out of the four Mono/Macro subpopulations identified in our study, including TAMs, AM_2, and AM_3 (Supplementary Fig. S2g–j). These macrophage lineages seemed to exhibit distinct tissue distributions. While AM_1 was composed exclusively of cells from NATs, and TAMs from the TME, AM_2 and AM_3 were composed of cells from both NATs and the TME, implying that they could be derived from precursors localized in lung tissues or potential migratory features of these two subtypes between tumor tissue and adjacent normal lung tissue.^27^ AM_2 exhibited higher expression levels of major histocompatibility complex (MHC) cluster II molecules (including *HLA-DQA2*, *HLA-DRB6*, and *HLA-DRB5*) than AM_1, implying enhanced antigen-presenting abilities of this cluster (Fig. 2h). AM_3 featured cell cycle-associated genes, such as *MKI67*, *AURKB*, *CDK1*, and *ASPM*, and had a similar gene expression patterns to AM_2 and TAMs, suggesting strong proliferative properties and the potential state transition ability of this cluster (Fig. 2h). In contrast, TAMs in the TME of SCLC expressed high levels of *TCF4*, *IL2RA*, *PLA2G7*, *GAL3ST4*, *PLTP*, *CMKLR1*, *ADAMDEC1*, and *MMP12* (Fig. 2h), suggesting an immunosuppressive feature,^28^ and thus providing potential targets for SCLC immunotherapies. *IDO1*, which is induced by inflammation within the TME and promotes a tolerogenic environment through immunosuppressive myeloid cell populations,^29^ was highly expressed in DCs in the SCLC TME (Supplementary Fig. S2f). Our single-cell data revealed diverse immune cell states of the SCLC microenvironment and may help to better understand and develop novel treatment strategies for SCLC.

### Heterogeneity of SCLC malignant cells

**Fig. 3:**
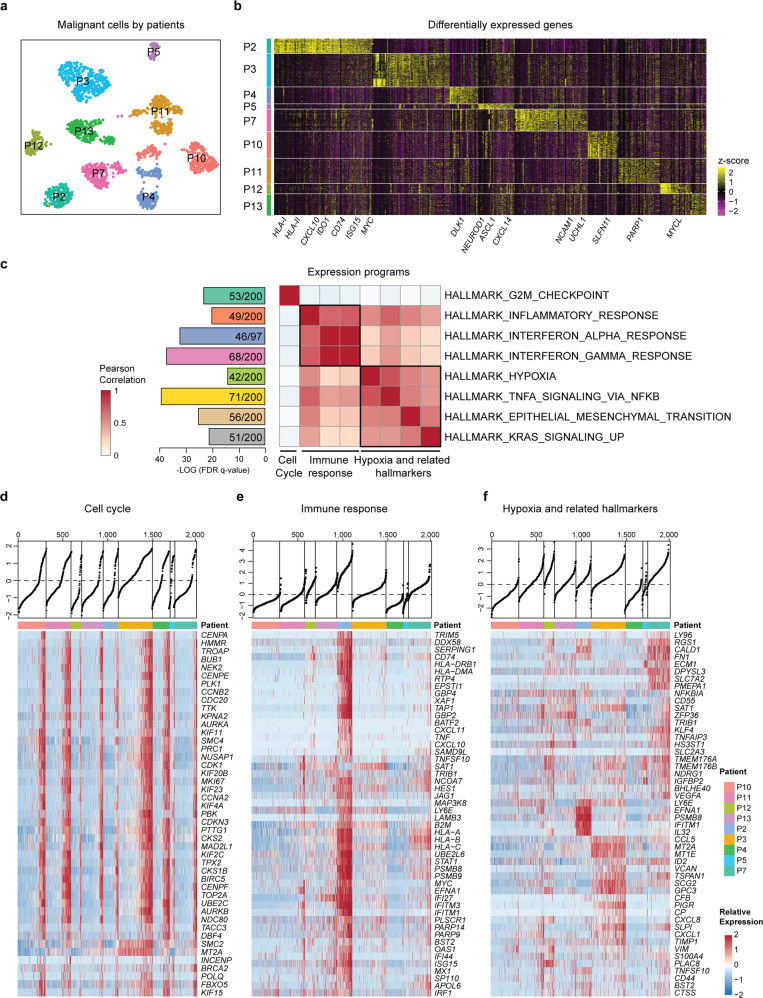
Expression heterogeneity of the malignant cell compartments in primary SCLC. **a** UMAP plot of malignant cells from nine SCLC patients (indicated by color) reveals tumor-specific clusters. **b** Heatmap of DEGs (top 100 genes in each patient, ranked by the log fold-change in the average expression between cells from one patient and all other cells) across nine individual SCLC primary tumors (columns). Expression data were normalized with z-score transformation, in which yellow and purple represent the high and low expression of a gene, respectively, relative to the median expression level (see the color scale). Selected genes are highlighted. MHC class I molecules (MHC-I) mainly included *HLA-A/B/C/E/F*; MHC class II molecules (MHC-II) included *HLA-DMA/PA1/RA/B1/B5*. **c** GSEA of DEGs (top 2,000 genes ranked by their dispersion values) using hallmark gene set collections. The most enriched hallmarks (ranked by the FDR *q*-values) are shown with the number of mapped and total genes in the pathways. Pearson correlation analysis was performed based on the mean expression levels of the genes involved in these hallmarks. **d** Heatmap reveals the expression of the proliferation program and representative genes from the program (rows) in individual SCLC cells (columns). Cells were grouped by tumor and ordered by the single-sample GSEA (ssGSEA) score (top). **e** Heatmap depicts the expression of the immune program (the inflammatory response, interferon α response, and interferon γ response) as shown in (**c**) and (**f**), Heatmap depicts hypoxia-related hallmarks (including TNFα signaling via NF-κB, EMT, *KRAS* signaling up, and hypoxia) as shown in **c**

To analyze the expression heterogeneity of the malignant compartment, we separated all malignant cells and ran UMAP. Although most cells were grouped by their tumor origins, representing extensive intertumor heterogeneity, many tumors contained separate subclusters, indicating significant ITH at the transcriptome level (Fig. 3a and Supplementary Fig. S3a). The DEGs mainly included those that respond to immune stimulates, such as MHC class molecules, *CD74*, *IDO1*, and *ISG15*, suggesting a close interaction between malignant and immune cells in the TME in this patient (SCLC-P2) (Fig. 3b, Supplementary Fig. S3b and Supplementary Table S3). In addition, key TFs related to SCLC tumor subtypes, such as *ASCL1*, *NEUROD1*, and *POU2F3*, were found to be differentially expressed in our cohort, as further described in the following sections. In addition, potential treatment targets or biomarkers for SCLC, such as *MYC*, *PARP1*, *SLFN11*, *CDK7*, *BCL2*, and *CD274*, were found to be differentially expressed in individual cells, highlighting the need for patient selection to improve targeted therapeutic strategies. Thus, the intertumor heterogeneity of malignant cell reflects differences in the interactions between TME components, SCLC subtypes, and different drug sensitivities.

To further explore what biological states or processes are involved among individual malignant cells, we performed GSEA by using the ‘Hallmark’ gene set collections for those genes with high variability.^30^ The DEGs were mainly involved in hallmarks such as the G2M checkpoint, inflammatory response, interferon α response, interferon γ response, TNFα signaling via NF-κB, EMT, *KRAS* signaling up, and hypoxia (Fig. 3c and Supplementary Table S4). Pearson correlation analysis revealed that these hallmarks could be categorized into programs associated with proliferation, the immune response and hypoxia-related hallmarks (Fig. 3c). The most notable feature of the module analysis was the activity of the proliferation program in a large proportion of cells from each tumor, ranging from 19.8% in SCLC-P12 to 75.4% in SCLC-P3 (Fig. 3d). The proliferation program included *MKI67*, which is a typical biomarker used in the clinic to indicate the proliferating properties of SCLC, and *TOP2A* (topoisomerase II), which is the target of etoposide that is most frequently used for SCLC treatment in combination with platinum (Supplementary Fig. S3c). Thus, the proliferation program was closely associated with clinically reported *MKI67* scores based on the immunohistochemistry (IHC) results (Supplementary Fig. S3d). Compared with the proliferation program, the immune expression patterns showed more pronounced intertumor heterogeneity in malignant cells from SCLC-P2 (Fig. 3e), which was in accordance with the enrichment of infiltrating immune cells. Reordering the cells by hypoxia-related hallmarks also revealed a gradient in each sample (Fig. 3f), indicating heterogeneous malignant components that might be associated with variable biological processes, such as angiogenesis, nutritional/blood supply, and tumor metastasis. Here, we further validated the ITH of these diverse transcriptional programs with published single-cell data (Supplementary Fig. S3e–g).^18^ Together, these data revealed a recurrent feature of tumor cell heterogeneity in SCLC (i.e., the expression of diverse transcriptional programs), which has great potential for better understanding the SCLC biology, optimizing current treatment, and developing new therapeutic strategies for SCLC.

### Molecular subtype heterogeneity of SCLC

**Fig. 4:**
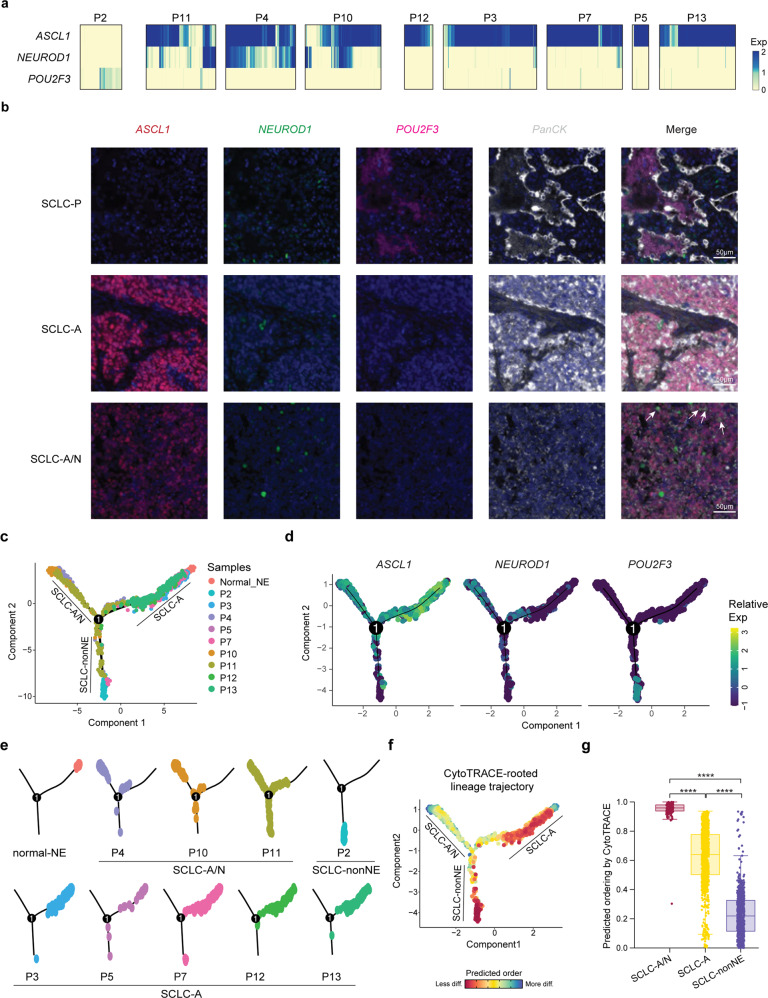
Intertumor heterogeneity and ITH of the molecular subtypes of SCLC defined by the expression of key TFs. **a** Expression of *ASCL1*, *NEUROD1* and *POU2F3* in individual cells from nine patients. **b** Multiplex immunofluorescence imaging shows the expression patterns of *ASCL1* (red), *NEUROD1* (green) and *POU2F3* (pink) in SCLC samples at the protein level, in accordance with the expression patterns of key transcriptional regulators. PanCK (gray) was used to stain epithelial cells in SCLC tumors. DAPI was used to label cell nuclei. Scale bars, 50 µm. White arrows indicated co-expression of *ASCL1* and *NEUROD1*. **c** Pseudotime trajectory of malignant SCLC cells in a two-dimensional state space inferred by Monocle 2. Monocle components were correlated with the SCLC-A, SCLCA/N and SCLC-non-NE clusters. Each dot corresponds to one single cell, colored according to its patient of origin. **d** Relative expression of *ASCL1*, *NEUROD1* and *POU2F3* in three trajectory branches. **e** Faceted pseudotime plots indicating the distribution of cells from each patient. **f** Combined application of CytoTRACE and Monocle 2 to dissect SCLC differentiation. diff, differentiated. **g** Boxplots showing CytoTRACE values for three Monocle branches in (**f**). Statistical analyses were performed using the Wilcoxon signed-rank test. ****p-value < 0.0001

Although SCLC is considered as a molecularly homogeneous malignancy, recent analyses have led to the classification of molecular subtypes based on the intertumor heterogeneity of *ASCL1*, *NEUROD1*, *POU2F3*, and *YAP1* expression: these subtypes are termed as SCLC-A, SCLC-N, SCLC-P and SCLC-Y.^9^ SCLC-A and SCLC-N are considered to be SCLC with neuroendocrine characteristics (SCLC-NE), in which SCLC-A represents the ‘classic’ subtype of SCLC-NE and SCLC-N represents the ‘variant’ subtype. Although the population-level data revealed the dominant transcriptional program, we continued to explore whether individual cells in a tumor could vary according to these classifications. Our data revealed that *ASCL1*, *NEUROD1*, and *POU2F3* were exclusively expressed in the malignant cells of SCLC tumors, however, *YAP1* was mainly expressed in normal epithelial cells rather than tumor cells (Supplementary Fig. S4a). We then determined the subtypes of our nine SCLC tumors on the basis of merged single-cell RNA expression data. Of note, most tumors clearly mapped to one of the four subtypes: SCLC-A (*n* = 5) and SCLC-P (*n* = 1) (Supplementary Fig. S4b). None of the malignant cells mapped to the SCLC-N or SCLC-Y subtypes, probably due to their low frequencies among SCLC subtypes.^9^ Strikingly, three patients (P4, P10, and P11) expressed high levels of *ASCL1* and variable levels of *NEUROD1*; this was termed the SCLC-A/N subtype in our study (Supplementary Fig. S4b). We then examined the expression of *ASCL1*, *NEUROD1* and *POU2F3* in individual malignant cells across tumors. All five SCLC-A tumors consisted of cells that expressed uniformly high levels of *ASCL1*, conforming to their bulk-level subtypes. However, a small number of cells exhibited highly divergent gene expression patterns; for example, low levels of both *ASCL1* and *NEUROD1*, represented the non-neuroendocrine (non-NE) subpopulation in SCLC-A tumors (Fig. 4a). In contrast, all three SCLC-A/N tumors consisted of heterogeneous malignant cells corresponding to different SCLC subtypes (SCLC-A, SCLC-A/N, SCLC-N and SCLC-non-NE) (Fig. 4a). The SCLC-P tumor, with no detectable expression of *ASCL1* and *NEUROD1*, consisted of individual cells expressing heterogeneous levels of *POU2F3*, with half of the cells having no detectable expression of *POU2F3* (Fig. 4a). Surprisingly, the SCLC-non-NE cells in SCLC-A and SCLC-A/N and some SCLC-P tumors expressed neither *POU2F3* nor *YAP1*, which might suggest new subtypes dominated by other TFs or the existence of transitional states (Supplementary Fig. S4c).^31^ To understand the spatial relationships of these transcriptional states, we performed multiplex immunofluorescence staining of these three TFs in all the samples. These images confirmed the presence of multiple subtypes within these tumors in proportions similar to those identified by scRNA-seq analysis (Fig. 4b). These single-cell results confirmed the diversity of tumor cell states in SCLC tumors and the regulatory functions of *ASCL1*, *NEUROD1* and other TFs that shape the states of malignant cells in SCLC patients.

To better understand the transcriptional relationships among the malignant cells, we applied an unsupervised inference method, Monocle 2, to construct the potential transitional trajectories. Unsupervised pseudotime suggested a branched trajectory, with SCLC-A/N and SCLC-non-NE cells positioned at the opposite branches from SCLC-A cells (Fig. 4c, d and Supplementary Fig. S4d). The faceted pseudotime trajectory revealed that the SCLC-A and SCLC-A/N branches were mainly composed of cells from SCLC-A tumors and SCLC-A/N tumors, respectively (Fig. 4e and Supplementary Fig. S4e). In contrast, the SCLC-non-NE branch included cells mainly from P2 and some non-NE cells from SCLC-A/N tumors. As it has been widely accepted that SCLC derived from pulmonary NE cells in the lung expresses *ASCL1*, we included 54 normal neuroendocrine (normal NE) cells described by Vieira Braga, F.A., et al.^32^ As expected, normal-NE cells were located at the start of the SCLC-A branch, and an evolutionary trajectory from SCLC-A to other subtypes was indicated (Fig. 4c, e, Supplementary Fig. S4e). To further determine the ‘roots’ and developmental trajectories of SCLC, we applied CytoTRACE to delineate cellular hierarchies based on the number of expressed genes per cell.^33^ The results indicated two possible ‘roots’ with fewer differentiation states, one of which was at the start of the SCLC-A branch (Fig. 4f). Derived from normal NE cells that have a high degree of differentiation, SCLC-A cells exhibited a high degree of dedifferentiation, while SCLC-A/N cells showed an increased degree of differentiation, suggesting that SCLC-A/N might be derived from other SCLC cells (Fig. 4g). The other potential ‘roots’ were at the SCLC-non-NE branch, which was mainly composed of cells from P2, indicating that this SCLC-P tumor could arise from a distinct cell of origin (not normal NE cells) (Fig. 4f, g). As *POU2F3* is a master driver of tuft cells, a rare chemosensory cell type in the pulmonary epithelium,^9^ our findings suggested that SCLC-P tumors might arise from tuft cells,^34^ but further validation is needed.

Together, these results confirmed the rationality of partitioning by using these key TFs as a fundamental feature of the molecular landscape of SCLC. Compared with the representation of SCLC subtypes at the bulk level, our scRNA-seq data revealed the representation of multiple SCLC subtypes at the single-cell level, at varying ratios, in most (or all) SCLC tumors, which emphasizes the importance of applying single-cell sequencing and signals an urgent need for functional studies on ITH with respect to progression and treatment.

### Functional associations of intratumor subtype signatures

**Fig. 5:**
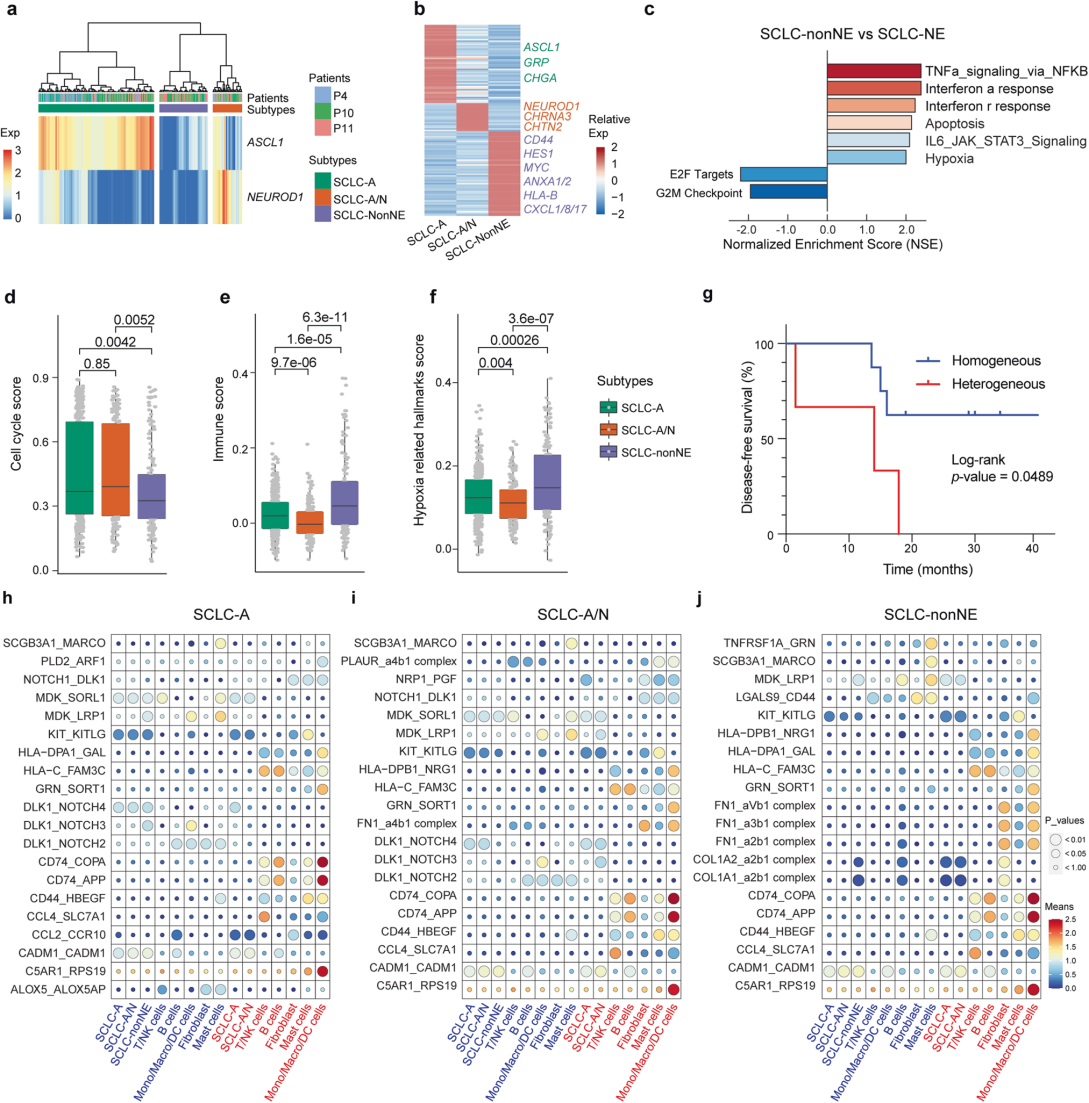
Inter/intratumor heterogeneity of the subtypes of SCLC defined by the expression levels of *ASCL1* and *NEUROD1*. **a** Hierarchical clustering of *ASCL1* and *NEUROD1* expression in malignant cells from P4, P10 and P11 revealed three main clusters. **b** Heatmap shows the DEGs among the three clusters, with interesting genes indicated in corresponding colors. **c** GSEA was performed to interpret gene expression data from the SCLC-non-NE cluster compared with the SCLC-NE cluster (including SCLC-A and SCLC-A/N). **d**–**f** Scores of the three subtypes regarding the cell cycle- (**d**), immune- (**e**) and hypoxia-related (**f**) hallmarks described in Fig. 2. The boxes display the interquartile range (IQR = Q3–Q1; the 25th (Q1) to the 75th percentiles (Q3)), with the centerline denoting the median. Whiskers were drawn to represent Q1 minus 1.5 × IQR and Q3 minus 1.5 × IQR. All other observed points are plotted as outliers. Dots were added to box plots using the function geom-dotplot. Statistical analyses were performed using one-way ANOVA followed by Bonferroni’s multiple comparisons test, with the *p*-values indicated. **g** Disease-free survival (DFS) was analyzed by the comparison of 3 SCLC patients (heterogeneous) to 6 other SCLC patients (homogeneous). The log-rank test was performed to indicate a difference with a *p*-value of less than 0.05. **h**–**j** Dot plots derived from CellPhoneDB show selected ligand–receptor interactions (top 20 based on the expression level) from SCLC-A (**h**), SCLC-A/N (**i**) and SCLC-non-NE (**j**) clusters with other clusters or cell types. The size of the circle represents the *p*-values. The means of the average expression level of interacting pairs are indicated by color. The cell clusters labeled blue and red on the ‘*x*’-axis indicate that they act as receptors and ligands in the interaction pairs, respectively

Although a non-NE NOTCH-active small-cell lung cancer cell subpopulation has been described in the genetically engineered mouse models (GEMMs),^35^ its function and significance in human tumors remain unclear. We next explored the functional associations of intratumor subtype signatures across individual cells, focusing on three SCLC-A/N tumors in which the largest numbers of heterogeneous cells were included (P4, P10, and P11). Based on the expression of *ASCL1* and *NEUROD1*, malignant cells were clustered into three subtypes: SCLC-A, SCLC-A/N (SCLC-N included), and SCLC-non-NE (Fig. 5a, Supplementary Fig. S5a). Malignant cells from both the SCLC-A and SCLCA/N subtypes expressed genes such as *GRP*, *UCHL1* and *CHGA* (Fig. 5b), displaying typical neuroendocrine features of SCLC. In contrast to SCLC-A cells, the expression of *NEUROD1* in SCLC-A/N cells did not result in significant gene expression alterations, with the DEGs mainly including those associated with neural development, such as *NEUROD1*, *CHRNA3*, and *CNTN2* (Fig. 5b). In SCLC-non-NE cells, a number of genes, such as *MYC*, *CD44*, *HES1*, *ANXA1/2*, and *CXCL1/8 /17*, were highly expressed. *MYC*, which has been demonstrated in GEMMs to drive a NE-low “variant” subset of SCLC with *NEUROD1* expression,^10^ exhibited a gradually decreased expression pattern from SCLC-non-NE to SCLC-A/N to SCLC-A cells (Supplementary Fig. S5b). Recent studies have suggested that *MYC*-amplified SCLC may be sensitive to aurora kinase inhibitor (alisertib) and *CHK1* inhibitors,^10^ suggesting a combinational strategy with drugs that target SCLC-NE populations for SCLC treatment. *CD44*, a multifunctional cell surface adhesion receptor, has been described to be highly expressed in non-NE SCLC cells,^35^ and is associated with the migration and invasion processes involved in the metastasis of this subtype (Supplementary Fig. S5c).^36^ Consistent with the results from GEMMs and human SCLC, the high expression of *HES1* (Supplementary Fig. S5d), a transcriptional target of the NOTCH signaling pathway, together with the low expression of *DLK1* (Supplementary Fig. S5e), indicated an activated NOTCH signaling pathway in this cell population. To target the heterogeneous compositions of SCLC-NE and SCLC-non-NE malignant cells, we developed a combinational treatment strategy that composed of etoposide and cisplatin (EP), a conventional chemotherapy for SCLC, in combination with silibinin (a major bioactive component of the plant *Silybum marianum*) that has been studied extensively for its efficacy in cancer by inhibiting *CD44* promoter activity.^37,38^ The addition of silibinin greatly enhanced the cancer inhibition efficacy of chemotherapy for SCLC both in vitro and in vivo (Supplementary Fig. 5f–h).

We next performed GSEA to explore the biological functions associated with these three clusters. Compared with SCLC-non-NE cell populations, SCLC-NE (including SCLC-A and SCLC-A/N) cell populations exhibited prominent enrichment of signatures of the hallmarks of E2F targets and the G2M checkpoint (Fig. 5c and Supplementary Fig. S6a), indicating a relatively strong proliferation signature in this cell population. The co-expression of *NEUROD1* with *ASCL1* might drive malignant cells to a ‘variant’ subtype of SCLC, showing an enrichment of signatures involved in the hallmarks of angiogenesis and EMT (Supplementary Fig. S6b), which would promote tumor cell survival and metastasis.^39^ This ‘hybrid’ state might indicate biological plasticity between subtypes that is shaped by the microenvironment and tumor progression. In contrast, the SCLC-non-NE clusters with relatively low proliferation ability exhibited prominent enrichment of signatures involved in the hallmarks of TNFa signaling via NF-κB, the interferon α response and the interferon γ response, indicating a close interaction with immune components in the SCLC microenvironment (Fig. 5c and Supplementary Fig. S6c).

To further describe the heterogeneous characteristics of these subtypes, we analyzed the gene expression scores of cell cycle-, immune-, and hypoxia-related hallmarks that were described in the previous section. In accordance with the GSEA results, cells from the SCLC-NE subtype, including SCLC-A and SCLC-A/N, exhibited a higher cell cycle score, while the SCLC-non-NE subtype exhibited the highest immune- and hypoxia-related hallmark scores (Fig. 5d–f, Supplementary Fig. S6d–f). The ITH of SCLC inferred by TF expression was associated with a short disease-free survival (DFS) time after surgical treatment in our study (Fig. 5g). In addition, we also performed immunohistochemistry (IHC) on formalin-fixed paraffin-embedded (FFPE) tissues from a large cohort of 90 SCLC patients (Supplementary Table S5), in which, the expression of *ASCL1* and *NEUROD1* were evaluated at the protein level (Supplementary Fig. S6g). The results further indicate that heterogeneous expression of *ASCL1* and *NEUROD1* is associated with a relatively short overall survival (OS; Supplementary Fig. S6h). Using CellPhoneDB, we illustrated the interacting molecules of ligand–receptor pairs and confirmed that SCLC-non-NE clusters tended to have more interactions with other cell clusters and immune and stromal cells (Supplementary Fig. S6i, j). While *CD74_COPA*, *CD74_APP* and *C5AR1_RPS19* were significantly enriched in all three SCLC clusters with macrophages/monocytes/DCs, specific pairs were enriched in different SCLC clusters (Fig. 5h–j, Supplementary Table S6).

In brief, although population-level data revealed the dominant transcriptional programs, our single-cell transcriptional data revealed intratumor subtype heterogeneity, potentially providing important insights into SCLC tumor biology and clinical implications for SCLC treatment.

### ITH of SCLC is recapitulated during tumor relapse

**Fig. 6:**
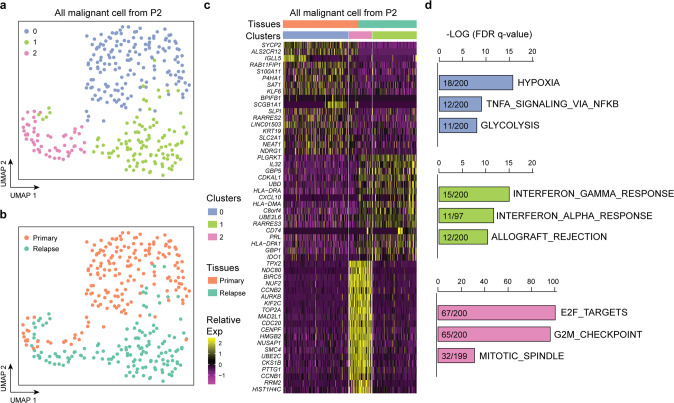
ITH of SCLC is recapitulated in relapsed tumors. **a** UMAP plot of malignant cells from SCLC-P2 revealed three specific clusters (indicated by color). **b** UMAP plot of malignant cells from SCLC-P2 revealed specific clusters in the primary and relapsed tumors by the cell of origin. **c** Heatmap shows the DEGs and related pathways that correspond to the primary and relapsed tumors, as well as the three specific clusters. **d** GSEA of all DEGs using hallmark gene set collections. The top 3 enriched hallmarks (ranked by the FDR *q*-values) along with the number of mapped and total genes in the pathways are shown

To gain further insight into potential determinants of SCLC relapse, we closely monitored these patients for over three years and ultimately obtained one fine-needle biopsy sample after relapse from SCLC-P2. While most cell clusters contained cells from both the PT and RT, most cells from the NAT were identified as myeloid cells (Supplementary Fig. S7a, b). We then isolated malignant cells from both the PT and RT. UMAP revealed three clusters, two from the PT and one from the RT (Fig. 6a, b), and an overlapping cluster composed of cells from both origins. DEG analysis and GSEA revealed that the PT cells exclusively expressed immune-associated genes and hallmarks, while the RT cells mainly expressed hypoxia- and apoptosis-associated genes and pathways (Fig. 6c, d). The consistent signature between the PT and RT were mainly enriched in cell cycle-associated hallmarks, such as E2F_TARGETS, G2M_CHECKPOINT, and MITOTIC_SPINDLE, representing a subpopulation in both the PT and RT with high proliferation activity. *POU2F3* expression was consistent during tumor relapse (Supplementary Fig. S7c), with a consistent expression level in some of the malignant cells from both the PT and RT. To gain further insight into potential determinants of SCLC recurrence, we deduced the CNVs in the PT and RT of P2 (Supplementary Fig. S7d). The RT exhibited CNV patterns similar to those of the PT, including the deletion of 3p, 4p, 4q, and 10q, the deletion of 13q (containing *RB1*) and 16q, and the loss of 17p (containing *TP53*). Our results indicate that although there were no significant genomic-level alterations during tumor recurrence, transcriptional evolution does occur during tumor relapse.

### SCLC subtypes associated with different immune microenvironments and therapeutic responses

**Fig. 7:**
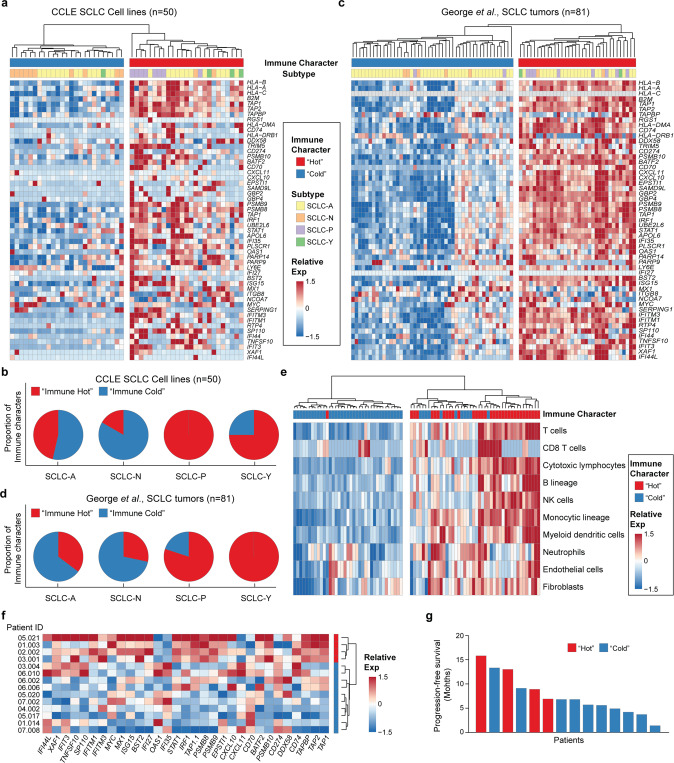
SCLC subtypes associated with different immune microenvironments. **a**, **b** Heatmap of gene expression according to the immune signatures shown in Fig. 3e in 50 SCLC cell lines from the CCLE (**a**) and 81 SCLC tumor samples described by George et al. (**b**). The immune characteristics of “hot” and “cold” tumors, as well as SCLC subtypes, are indicated. **c**, **d** Pie charts illustrate the proportion of different immune characteristics in SCLC subtypes from 50 SCLC cell lines from the CCLE (**c**) and 81 SCLC tumor samples described by George et al. (**d**). **e** Qualitative and quantitative analyses of the abundance of eight immune and two stromal cell populations in 81 SCLC tumor samples described by George et al. **f** Heatmap of gene expression according to the immune signatures shown in Fig. 3e in 14 SCLC patients who received ICB treatment. **g** Progression-free survival (PFS) of these 14 SCLC patients. The immune “hot” (red) and “cold” (blue) characteristics are indicated

The heterogeneous expression patterns of immune-associated properties by malignant cells as well as immune components suggested that SCLC might have different immune microenvironments that are associated with distinct subtypes, which prompted us to extract additional insights from bulk SCLC data. First, we downloaded the bulk expression data for 50 SCLC cell lines from the Cancer Cell Line Encyclopedia (CCLE) and categorized them according to the key TFs *ASCL1*, *NEUROD1*, *POU2F3* and *YAP1* (Supplementary Fig. S8a). The immune signature of these cell lines revealed a heterogeneous intertumor immune profile, with half (25/50) of the cell lines expressing strong immune characteristics (Fig. 7a), which were termed as ‘immune hot’, and half expressing weak immune characteristics (‘immune cold’). “Immune hot tumors” refer to immune cell well-infiltrated tumors, whereas “Immune cold tumors” refer to immune cell poorly- or non-infiltrated tumors, which is associated with immunotherapy responses in many cancer types.^40^ Our data also revealed that most SCLCs with NE characteristics, including SCLC-A and SCLC-N, tends to have a ‘immune cold’ character, while non-NE SCLCs, including SCLC-P and SCLC-Y, tend to have ‘immune hot’ characteristics (Fig. 7b). To validate these results, we further downloaded the bulk expression data from 81 human SCLC tumors described by George et al.,^8^ including data from malignant cells, stromal cells, and infiltrating immune cells. The relative frequencies of these four subtypes were similar to those of the CCLE cell lines and in accordance with those described in the review by Rudin et al.,^9^ with the SCLC-A as the most frequent subtype (Supplementary Fig. S8b). The data also revealed that most the SCLC-NE tumors exhibited cold immune characteristics (45/69; 65%) (Fig. 7c), and non-NE SCLC tumors tended to exhibit hot immune characteristics (10/12; 83%) (Fig. 7d). To investigate whether changes in immune and stromal cell type components in the TME are correlated with SCLC tumors with different immune characteristics, we used the MCP-counter method to deduce the relative abundance of heterogeneous cell populations based on bulk gene expression data. The results indicated that SCLC tumors that exhibited the hot immune signature were associated with more immune and stromal cell infiltration (Fig. 7e).

To further deduce whether SCLC tumors with different immune properties would be associated with different immune checkpoint blockade (ICB) responses, we performed targeted gene expression analysis of 14 samples from SCLC patients who received anti-*PD-1* treatment. Based on our immune genes, four patients were identified as having ‘immune hot’ features, while the other patients (n = 10) were identified as having ‘immune cold’ features. With close follow-up for 20 months, our data indicated that patients with ‘immune cold’ SCLC tended to benefit more from ICB than patients with “immune hot” SCLC. These findings suggest significant intertumor heterogeneity and ITH of SCLC regarding immune characteristics and warrant validation in large cohorts.

## Discussion

Research on SCLC has been hampered by a lack of available tumor tissues due to disease aggressiveness, thus limiting our understanding of the landscape of the SCLC TME and possibly constituting one of the reasons behind the failure of current therapies. Although surgical resection is rarely a therapeutic option for SCLC, our study included 11 matched samples of PTs and NATs. Here, by using a modified STRT-seq approach, we presented a comprehensive characterization of this disease. Our study revealed a complex cellular ecosystem of the SCLC tumor microenvironment, including the heterogeneous characteristics of malignant cells and tumor-infiltrating immune and stromal cells. As SCLC has historically been regarded as a single disease and the standard treatment for SCLC has not been improved for decades, our study revealed a previously unappreciated cellular architecture of SCLC at single-cell resolution, thus providing fundamental implications to SCLC biology and therapeutic strategies.

While intertumor heterogeneity mainly describes the differences across individual patients, intratumor heterogeneity (ITH) refers to the variations among individual cells and a complex network of extracellular matrix (ECM). A better understanding of the numerous mechanisms of both tumor heterogeneities has been achieved along with advanced molecular and biochemical technologies.^41^ Both intrinsic and extrinsic factors, such as genetic and epigenetic alterations, gene expression switching, and influences of surrounding microenvironment, contribute to ITH.^42^ In our study, by performing multiregional analyses at single-cell level, we interrogated ITH. First, the inferred CNVs revealed significant spatial ITH of SCLC at genomic level, which might also contribute to the differential transcriptomic profiles of SCLC. Second, we deduced the ITH at both biological hallmarks of malignant cells as well as subtypes based on the expression of key transcription factors of SCLC. Meanwhile, we revealed the associations of these two ITH properties and the interactions with tumor infiltrating immune cells. Third, by including normal cells from adjacent normal lung tissues, we were able to deduce the origin of the tumor cells by analyzing trajectory paths to specific malignant subtypes, which could be regarded as one of the most important causes of SCLC ITH.

Our data revealed the existence of heterogeneous expression programs of malignant cells related to the cell cycle, immunity, hypoxia, EMT and other hallmarks. The cell cycle program is present in a large proportion of all SCLC patients, explaining why SCLC patients always benefit from the radiation therapy and chemotherapy that target these highly proliferating cell populations.^43^ Other relatively quiescent cells with diverse transcriptional programs, which might drive therapeutic resistance and tumor relapses,^44^ provide actionable targets for combinational treatment strategies with current therapy regimens.^36^ We also observed that the cell cycle program consistently existed in longitudinal samples of PTs and RTs after surgical treatment, while other programs experienced state switching or evolution during tumor relapse.

SCLC has been categorized into subtypes based on the expression of key transcriptional regulators at the bulk level. However, the classification and characterization of SCLC subtypes are still in progress, especially at single-cell resolution. Our scRNA-seq results revealed the co-expression or mutually exclusive patterns of key TFs, most commonly *ASCL1* and *NEUROD1*. Our data also revealed a cell population in nearly all SCLC tumors with varying ratios that expressed none of these four TFs, implying plasticity between subtypes or even from different cell origins.^45–47^ The results also suggested that there might be exceptions of SCLC cases at the bulk level, despite rare, that are beyond the current subtypes and dominated by other TFs and thus need to be further specified. Deep mining of the ITH of diverse subtypes enables us to identify the associated biomarkers and signaling pathways, paving the way for optimizing current treatments and developing novel combinational therapeutics.

The malignant cells in NE tumors in our study exhibited uniform immune features, highlighting their potential of interactions with immune components and a distinct microenvironment.^48^ We then validated the results by analyzing SCLC cell lines from the CCLE as well as bulk transcriptome data described by George et al.^8^ As expected, approximately half of the CCLE cell lines and SCLC cases in the George dataset, most of which were non-NE SCLC, exhibited strong immune properties (Fig. 7). In addition, as the immune properties were calculated based on the immune-associated markers that expressed on malignant cells, high levels of immune cell and stromal cell infiltration were observed in these SCLC tumors. Our scRNA-seq data, together with public transcriptomic data from a large number of SCLC tumors, revealed the ‘immune desert’ and ‘immune oasis’ phenotypes of SCLC and strong correlations with NE/non-NE characteristics.^49^ These immunology subtypes might be correlated with the clinical outcomes of immunotherapy and be used to help select patients who might benefit from these promising strategies.^50^

In a recent study on SCLC by JM Chan et al., scRNA-seq technology was used to demonstrate a certain level of biological complexity in SCLC. However, although 21 SCLC patients were included, a substantial number of samples was collected from small biopsies, which may not fully represent the biology of the entire tumor. In addition, the study cohort included variable samples with a diversity of treatment histories, tissue locations, and SCLC subtypes. In comparation, our study mainly included SCLC patients with more tightly restricted clinical variables. First, we only included surgically resected samples (11/11), mainly from those patients who did not receive any neoadjuvant therapy (9/11), so as to better recapitulate the original landscape of the SCLC TME. Second, we included paired adjacent normal lung tissue and primary tumors, providing valuable comparisons to unravel the unique characteristics of the SCLC TME. Third, to better decipher the role of ITH in SCLC, we performed multi-region sampling of tumor tissues (2–4 regions per tissue), which were used to extensively delineate ITH by bulk-level sequencing. In addition to the unique sample cohort, our study presents two aspects of the ITH of SCLC: 1) the heterogeneous expression in malignant cells of genes related to the cell cycle, immunity, hypoxia, EMT, and other hallmarks; 2) the heterogeneous expression of key TFs of SCLC, most commonly *ASCL1* and *NEUROD1*. These results markedly enhanced our understanding of the clinical features of SCLC. Deep mining the ITH of SCLC is expected to enable the optimization of the current treatments and develop novel therapeutics. Of note, the results of our in vitro and in vivo experiments demonstrate that *CD44*, which was highly expressed in SCLC-non-NE populations, which are present to some degree in nearly all SCLC tumors, may be targeted to enhance the treatment efficacy of chemotherapy (cisplatin plus etoposide) for SCLC.

As one of the pilot studies that employ single-cell sequencing technologies to delineate ITH of SCLC, there are still some limitations. The sample size of our study is relatively small, including only one matched primary and relapsed tumor, which limits the scope of our analysis. To address this issue, we have included scRNA-seq data of 21 SCLC patients from the study by JM Chan et al. as validation. In addition, more in vitro and in vivo studies will contribute to further deciphering the relationship between ITH and clinicopathological features of SCLC. In fact, most of the conclusions obtained from our samples have been validated by using published data, a validation cohort composed of patients from different platforms (Cancer Hospital, Chinese Academy of Medical Sciences and Peking Union Medical College vs Memorial Sloan Kettering Cancer Center (MSKCC)) and generated with different sequencing methods (STRT-seq vs 10X Genomics). The fine-needle biopsy also gave limited information about the tumor. While the collection of more paired surgical samples during SCLC relapse presents technical challenges in clinic, genetically engineered mouse models (GEMMs) of SCLC and the development of future technologies by using archival tissues may help to address the issues.^15^ In summary, our study provides an unbiased high-precision scRNA-seq analysis of the TME in human SCLC, serving as the basis for directing the design of therapies.

## Materials and methods

### Patients and tumor specimens

In total, 11 patients who were histologically diagnosed with early-stage SCLC and underwent surgical resection between July 2017 and April 2019 were included in our study. Of these patients, two (SCLC-P1 and SCLC-P14) received inductive chemotherapy (etoposide plus cisplatin), while the other 9 did not receive therapy (treatment-naïve). Primary tumor (PT) tissues and paired adjacent normal lung tissues were collected immediately after surgical resection. For each PT tissue, we performed multi-region sampling (2–4 regions for each patient) according to the sample size. Each region was divided into two parts: one for single-cell collection and the other for hematoxylin and eosin (H&E, to obtain accurate pathological diagnosis information for each sample site) and multiplex immunofluorescence staining. With a close follow-up of over 2–4 years after surgery, 6 patients relapsed, and fresh fine-needle aspiration biopsy tissue from one of them was obtained and subjected to high-precision single-cell transcriptome analysis. Relevant clinical information, such as stage, tumor size, smoking status, treatment information, and disease-free survival, was collected (Supplementary Table S1). This study also included 14 patients who were diagnosed with extensive-stage SCLC and received first-line anti-PD-1 monotherapy (nivolumab) (Supplementary Table S2). Fine-needle aspiration biopsy tissues from treatment-naïve patients were obtained and subjected to targeted transcriptomic sequencing. We also analyzed publicly available transcriptome data of human primary SCLC tumor samples (*n* = 81, George et al., 2015)^8^ and human SCLC cell lines (*n* = 52, CCLE).^45^ This study was approved by the Ethics Committee of the National Cancer Center (NCC1799). All patients provided written informed consent to participate.

### Preparation of single-cell suspensions

Individual human SCLC samples were immersed in RPMI-1640 medium (Thermo Fisher Scientific, 21875034) immediately after resection. Within an hour, the fresh tissues were washed with cold phosphate buffered saline (PBS), dissected, minced, and transferred into digestion buffer ((2 mg/ml collagenase type I (Gibco, 17100017), 0.8 mg/ml Dispase II (Millipore, SCM133) and 0.2 mg/ml DNase I (Roche, 10104159001)). After incubation on a thermomixer (1000 × rpm) at 37 °C for 30 min, cell suspensions were filtered through a 70 μm strainer, pelleted (500 × *g*, 5 min, 4 °C), resuspended in 1 ml of 1× red blood cell lysis buffer (Sigma, R7757) and incubated for 3 min at room temperature. The reaction was stopped by adding RPMI-1640 medium. The dissociated cells were pelleted again (500 × *g*, 5 min, 4 °C) and resuspended in PBS supplemented with 1% bovine serum albumin for single-cell isolation.

### Whole-genome sequencing (WGS) and targeted transcriptome sequencing

In total, we performed low-pass WGS for 27 multi-region samples from 11 patients (2–4 regions for each patient). Genomic DNA was extracted with a DNeasy Blood & Tissue Kit (QIAGEN, 69506). The DNA concentration was quantified with a Qubit instrument, and DNA qualitiy was assayed with a Fragment Analyzer. Approximately, 500 ng genomic DNA was sheared to approximately 300 bp by the Covaris S2 system. The purified DNA fragments were used for library construction using the KAPA Hyper Prep Kit. The prepared libraries were subjected to sequencing on the HiSeq 4000 platform with 150-bp pared-end reads.

Targeted transcriptome sequencing was performed on the 14 fine-needle aspiration biopsy tissues. A custom RNA panel of 1392 genes of interest was designed with the DesignStudio software tool and polymerase chain reaction (PCR)-based library preparation was performed. An Agilent 2100 bioanalyzer system was used to evaluate the integrity of the extracted RNA. Sequencing was performed by using the NextSeq 500 platform (Illumina).

### scRNA-Seq data processing, quality control, and batch effect adjustment

Raw scRNA-seq data were processed as previously described.^51–53^ The cell barcode and UMI information were inserted in Reads2, and cDNA sequences were inserted mainly in Reads1. First, raw reads were split by cell barcodes in Reads2, and UMIs from Reads2 were added to the header of Reads1. Low-quality base pairs, adapters, template switch oligos (TSOs) and polyA sequences were then trimmed from Reads1, and low-quality reads (*N* > 10%) and short reads (<37 bp) were removed. Next, ‘clean’ Reads1 were mapped to hg19 (downloaded from UCSC) with TopHat (version 2.0.12). Uniquely mapped reads were counted via HTseq,^54^ and counts from the same UMI were merged to obtain the transcriptional count of each gene from single cells. Cells with fewer than 1000 genes detected or 1000 UMIs were excluded from further analyses. In addition, cells among in the top 1% according to gene expression were also filtered as doublet cells.^55^ Finally, a total of 4,911 cells with high-quality reads were retained for downstream analyses. We used the k-BET (a robust and sensitive k-nearest neighbors (KNN) batch effect test) R package to perform statistical analysis of possible batch effects.^55^ k-BET evaluates the accordance of replicates based on Pearson’s chi square test. By using this method, we aimed to find the most similar cells between the batches, which were assumed to belong to the same cell type. The systematic differences between the KNN cells were then used to quantify the strength of the batch, which was used to scale the rest of the cells in the batches. In detail, k-BET was run on major immune cell types, including myeloid cells, B cells, and T cells, under default parameters. A control dataset with known significant batch effects was included to assist with data integration. The algorithm creates a KNN matrix and chooses 10% of the samples to assess batch-label distribution in the neighborhood. If the local batch label distribution is sufficiently similar to the global batch label distribution, Pearson’s chi square test does not reject the null hypothesis (that is, ‘all batches are well mixed’). The neighborhood size k is fixed for all tests. The lower the result of k-BET (i.e., the mean test rejection rate), the less bias is introduced by the batch effect.

### DEGs and pathway analyses

DEGs were accessed through the FindMarkers and FindAllMarkers functions in Seurat,^56^ in which, the default two-sided nonparameteric Wilcoxon rank-sum test with Bonferroni correction was performed. With this method, only significant genes with the adjusted *p*-value of less than 0.05 and avg_logFC over 0.5 were considered marker genes and subjected to pathway enrichment analysis. The DEGs in malignant cells per patient were visualized by using Seurat’s DoHeatmap function, and their expression value was normalized from −2 to 2 (Fig. 3b).

### Cell type determination

To define the major cell types from scRNA-seq data, we first ran Uniform Manifold Approximation and Projection (UMAP) in Seurat for dimension reduction. DEGs were then identified for each cluster as indicated before and the top-ranked DEGs (according to the *p*-value and avg_logFC) were carefully reviewed. Feature plots were generated based on the top 10–20 DEGs, followed by a manual review process. High expression of epithelial (*EPCAM*, KRT family genes), canonical immune (*PTPRC*, *CD3D*/*CD4/CD8A/FOXP3* for T cells, *NKG7* for NK cells, *CD79A*/*CD19* for B cells, *CD68/CD163/MRC1* for myeloid cells, and *MS4A2/CPA3* for mast cells) and stromal cell (*DCN/COL1A1/FAP* for fibroblasts) markers in certain clusters was considered a strong indication of the clusters representing the corresponding cell type.

### Inference of copy number variations (CNVs) from scRNA-seq data

Large-scale CNVs were inferred from scRNA-seq data by using the inferCNV tool (https://github.com/broadinstitute/inferCNV; v1.2.1). As previously described,^14,22,55^ initial CNVs were estimated by sorting the analyzed genes by their genomic locations and applying a moving average to the relative expression values, with a sliding window of 100 genes within each chromosome. Normal epithelial cells from normal adjacent tissues (NATs) in this dataset were used as a reference for CNV analysis. The true CNVs called from WGS were used to assess the performance of the CNVs inferred from scRNA-seq data. In addition to the criteria for cluster distribution and marker gene expression, the inferred CNVs were applied to validate the identities of malignant and nonmalignant cells in the TME.

### Gene set enrichment analysis (GSEA)

GSEA was applied to evaluate the enrichment of a prior defined sets of genes associated with particular biological processes. In detail, GSEA was performed by calculating the enrichment score (ES) of each gene set on the preranked gene lists between two different samples or clusters.^57^ The normalized enrichment score (NES) was then yielded by normalizing the ES of each gene set to account for the size of the set. We also calculated the false discovery rate (FDR) by comparing the tails of the observed and null distributions for the NES. The hallmark (referred to as ‘H’) gene sets were obtained from the Molecular Signatures Database (MSigDB; v7.3).

### Definitions of proliferation, immune and hypoxia scores

We first calculated the standard deviation (SD) of each gene in all malignant cells from 9 SCLC patients. The top 2,000 genes with the highest SD values were subjected to pathway analysis by mapping to the hallmark gene sets obtained from the MSigDB, in which hallmarks with overlapping genes that met the FDR criterion were obtained. We then calculated the Pearson correlation coefficient of the top eight hallmarks with the highest FDR values to categorize them into three biological processes: proliferation (hallmark of the G2M checkpoint), immune (hallmark of the inflammatory response, interferon alpha response, and interferon gamma response), and hypoxia-associated pathways and signaling (abbreviated hypoxia; hallmark of hypoxia, TNF signaling via NFκB, epithelial mesenchymal transition (EMT), and KRAS signaling up). We used the top 45 genes in each biological process category to define the proliferation, immune and hypoxia scores as the average expression of these genes after z-score normalization.

### Single-cell trajectory analyses

We performed an unsupervised pseudotemporal analysis by using Monocle 2 to examine the trajectory of malignant cells dominated by different TFs.^58^ DDRTree, a reversed graph embedding algorithm in Monocle 2, was used to project our scRNA-seq data into a reduced dimensional space and reconstruct the temporal and bifurcation structure of the datatype based on global gene expression levels. In the gene selection step, we used the ‘dpFeature’ approach to perform unsupervised analysis, in which there was no forehead knowledge of the Monocle 2 genes used as input. Reverse graph embedding was used to reduce the data’s space to one with two dimensions. Then, the trajectory in the reduced dimensional space was visualized, in which we set the root based on the expression of key TFs in SCLC (including *ASCL1* and *NEUROD1*). We used a jitter plot to determine which state corresponds to those TFs.

### CytoTRACE

We used CytoTRACE,^33^ a computational framework, to predict developmental potential based simply on the number of expressed genes per cell. Generally, this algorithm predicts cellular differentiation states from scRNA-seq data based on the negative correlation between the number of expressed genes per cell and transcriptional diversity. We used CytoTRACE as a complement to the trajectory analysis from Monocle to deduce the potential developmental directions.

### Cell-cell communication analysis

We applied CellPhoneDB,^59^ a computational framework based on a public repository of ligands, receptors and their interactions, to infer cell-cell communications between different SCLC clusters and immune and stromal cell subsets in the SCLC TME. Based on the expression of ligands and receptors from our scRNA-seq dataset, biologically relevant interacting ligand–receptor partners between two cell subsets were identified. The permutation test was used to estimate the enrichment of ligand–receptor interactions, and those with *p*-value of less than 0.05 were visualized using dot plots, as shown in Fig. 5h.

### Deconvolution of immune and stromal cells from bulk gene expression data

We used the Microenvironment Cell Populations (MCP)-counter method^60^ to robustly quantify the abundance of tumor-infiltrating immune and stromal cell populations from bulk gene expression data (Fig. 7e). The results were realized by using the MCP-counter R package, which implements the MCP-counter method and predicts the abundance of 10 cell populations (8 immune populations, endothelial cells and fibroblasts) from the transcriptional profiles of human tissues.

### In vitro and in vivo antitumor efficacy of silibinin

The human SCLC cell lines H1048 and H69 were from BeiGene (Beijing) Co.Ltd., and cultured in RPMI-1640 supplemented with 10% FBS and antibiotics (1% penicillin/streptomycin) as previously described.^44^ All cells were maintained in 5% CO_2_ at 37 °C. silibinin, cisplatin, and etoposide were obtained from Millipore Sigma (CAS No. 22888-70-6 15663-27-1, and 33419-42-0). Cells were treated with silibinin at final concentration of 25 µM, cisplatin of 5 µM, and etoposide of 1 µM.^61^

Five-week-old female BALB/c nude mice used in vivo experiments were purchased from Beijing Vital River Laboratories. Human SCLC cell line H1048 (1.0 × 10^6^) was subcutaneously injected in the posterior flank in a volume of 100 µL serum-free media. Animals were monitored regularly and euthanized when they exhibited signs of morbidity or when the size of the subcutaneous tumor required sacrifice. Tumor volume was measured with a caliper: tumor volume = 1/2(length × width^2^). The tumor-baring mice were randomly divided into different experimental groups. Mice were treated by intragastric administration with silibinin (20 mg/kg)^62^ and/or by intraperitoneal injection with etoposide and cisplatin (4 mg/kg cisplatin dissolved in 0.9% saline solution on day 1, and 12 mg/kg etoposide dissolved in 0.9% saline solution on days 1–3).^44^ All procedures were conducted following the procedures approved by the Committee on the Ethics of Animal Experiments of the Health Science Center of Peking University.

### Immunohistochemistry (IHC) of formalin-fixed, paraffin-embedded (FFPE) sections

In our study, IHC combined with tissue microarrays, including tissue from 90 SCLC patients (Supplementary Table 5), was performed to evaluate the expression of *ASCL1* and *NEUROD1* at the protein level. In detail, tissue cores (1.5 mm in diameter) were obtained from FFPE tumor samples, using a hollow needle, and then inserted into a recipient paraffin block in a precisely spaced array pattern. Sections (4-μm thick) from this block were cut, mounted on microscope slides and analyzed by IHC.

FFPE sections were deparaffinized and rehydrated by immersing the slides in BioDewax and Clear Solution I (Servicebio) for 15 min; BioDewax and Clear Solution II for 15 min; BioDewax and Clear Solution III for 15 min; 100% ethanol twice for 5 min; 85% alcohol for 5 min; 75% alcohol for 5 min, and finally rinsed with deionized H_2_O. Antigen retrieval was performed and endogenous peroxidase activity was quenched. The sections were then blocked with serum blocking reagents for 30 min at room temperature, and incubated with a primary antibody (Anti-*MASH1* [anti-*ASCL1*], abcam, ab211327; anti-*NEUROD1*, abcam, ab60704) overnight at 4 °C and then a secondary antibody (HRP-labeled goat anti-rabbit IgG, Servicebio, GB23303) for 50 min at room temperature. DAB color developing solution was then added after the sections were rinsed with wash buffer 3 times for 15 min, and stopped by rinsing the sections with water. The stained tissues were mounted with a nuclear counterstain to better visualize tissue morphology. The stained tissues were then dehydrated, mounted, and examined by light microscopy. The hematoxylin stains nuclei blue, while positive detection with DAB gives a brownish yellow. IHC analysis of *ASCL1* and *NEUROD1* was defined as positive if the sample had an H-score^63,64^ in the top 30 samples (Supplementary Table 5). “Heterogeneous” samples were those with double positive expression of *ASCL1* and *NEUROD1*, while “homogeneous” refers to those samples with single positive or double negative expression of the two markers.

### Histopathology and multiplex immunofluorescence

FFPE Sections (4-μm thick) were mounted and routinely stained with H&E for histopathological examination (Supplementary Fig. S1a). Multiplex immunofluorescence was applied to identify the expression patterns of key transcriptomic regulators of SCLC, including *ASCL1* (Abcam, ab211327), *NEUROD1* (Abcam, ab60704) and *POU2F3* (Novus Biologicals, NBP1–83966). Multiplex immunofluorescence staining was performed using a PANO 7-plex IHC kit (Panovue, Cat# 0004100100), as previously described.^65^ In brief, the FFPE sections were subjected to deparaffinization, rehydration, and antigen retrieval according to the protocol supplied by the manufacturer. After blocking, the sections were incubated with a primary antibody and then a secondary antibody (polymer HRP-anti-mouse/Rabbit IgG). Other primary antibodies were sequentially applied by repeating the previous procedures. Nuclei were stained with DAPI (Sigma-Aldrich, D9542) after all the human antigens had been labeled. Multispectral images were obtained by scanning the stained slides with the Mantra System (PerkinElmer, Waltham, Massachusetts, US) and analyzed using inForm image analysis software (PerkinElmer, Waltham, Massachusetts, US) (Fig. 4c).

### Single-cell RNA-seq library construction and sequencing

A single-cell RNA-seq library was constructed according to the STRT-seq protocol with slight modifications.^66^ In brief, single cells were transferred to 96-well plates containing prepared cell lysis buffer by mouth pipetting. After cell lysis, mRNA from single cells was immediately reverse transcribed into cDNA with Superscript II Reverse Transcriptase (Thermo Fisher Scientific, 18064071), during which an 8 nt cell barcode and 8 nt unique molecular identifiers (UMIs) were added to the cDNA from each cell. Second-strand cDNAs were synthesized and subsequently preamplified using KAPA HiFi HotStart Ready Mix (KAPA Biosystems, KK2602). Then, the cDNAs were pooled together and purified. The products were then fragmented with a Covaris S2 ultrasonicator (Thermo Fisher Scientific), and the 3′ ends of cDNAs were enriched for library construction using a KAPA HyperPrep Kit (KAPA, KK8504) and sequenced on an Illumina HiSeq 4000 platform with 150-bp paired-end reads by Novogene.

### Statistics and reproducibility

No statistical method was used to predetermine sample sizes. Box plots were generated using the boxplot function of the ggplot2 R base package under default parameters. Violin plots, hybrid box plots and kernel density plots were generated using the ggplot2 R package to compare the distributions of different groups. The distribution shape of the data was estimated by kernel density estimation, where wider sections represent a higher probability that members of the population will take on the given value, and the skinnier sections represent a lower probability. Bar plots, which were generated by using the ggplot2 and ggpubr R packages, show the mean ± standard error of the mean, with individual data points displayed. Dot plots were generated by using ggplot2 to show gene expression in each categorical group (cell type). The dot size and color represent the fraction of cells expressing a given gene in each category and the average number of cells expressing the given gene within each category, respectively. Within each category, the normalized gene expression score is averaged only over cells expressing the given gene, where the gene is considered expressed if its normalized gene expression score is greater than zero. Comparisons between two groups were performed using unpaired two-tailed t-tests. One-way analysis of variance (ANOVA) with Tukey’s multiple comparisons test was used for multiple group comparisons. All statistical analyses and presentations were performed using R.

## Supplementary information

Supplementary Material

Supplementary Fig. S1

Supplementary Fig. S2

Supplementary Fig. S3

Supplementary Fig. S4

Supplementary Fig. S5

Supplementary Fig. S6

Supplementary Fig. S7

Supplementary Fig. S8

Supplementary Table S1

Supplementary Table S2

Supplementary Table S3

Supplementary Table S4

Supplementary Table S5

Supplementary Table S6

## Data Availability

The Single-cell RNA-seq data and WGS raw data generated in this study have been deposited in the Genome Sequence Archive (GSA) database of the National Genomics Data Center (NGDC, https://bigd.big.ac.cn/) under the BioProject accession code: PRJCA006026.
