# Supplementary material for: Single-cell transcriptomic profiling reveals the tumor heterogeneity of small-cell lung cancer
Source: Signal Transduct Target Ther. 2022 Oct 5;7:346. doi: 10.1038/s41392-022-01150-4 (PMC9532437; doi:10.1038/s41392-022-01150-4)
Supplement: Supplementary file 1 — Supplementary Material [file 41392_2022_1150_MOESM1_ESM.docx]

**Single-cell transcriptomic profiling reveals the tumor heterogeneity of small-cell lung cancer**

**SUPPLEMENTARY FIGURE LEGENDS**

**Supplementary Fig. S1** Single-cell transcriptome profiling of SCLC. **a**, Histology of all SCLC samples analyzed in this study. **b**, Stacked bar plots reveal the distributions of cell types in individual patients. **c**, Expression quantiles of typical biomarkers for all cell types. **d**-**f**, Heatmap shows large-scale CNVs in malignant cells (rows) from P7, P10, and P11. Multiple regions are indicated as PTR1 (primary tumor region 1) and PTR2 (primary tumor region 2). CNVs deduced from the bulk sequencing data in each region are illustrated. The red frame indicates chromosome 5q. **g**, Disease-free survival (DFS) was analyzing by comparing SCLC patients with a heterogeneous CNV in 5q with 6 other SCLC patients with a homogeneous CNV in 5q. The log-rank test was performed to indicate a difference with a *p*-value of less than 0.05.

**Supplementary Fig. S2** The immune landscapes of SCLC. **a**, Heatmap of typical T cell and cytotoxic biomarkers. **b**, UMAP plot of all T cells from both NATs and the TME color coded by patient. **c**, Expression of markers of exhausted T cells. **d**, UMAP plot of all myeloid cells from NATs. **e**, UMAP plot of all myeloid cells from the TME. **f**, UMAP plot of *IDO1* expression in all myeloid cells. **g, i** UMAP plot showing the major lineages of myeloid cells (**g**) and monocyte/macrophage (Mono/Macro) (**i**). **h, j** Bubble heatmap showing expression levels of selected signature genes in myeloid (**h**) and Mono/Macro cells (**j**). Dot size indicates fraction of expression cells, colored based on normalized expression levels.

**Supplementary** **Fig. S3** Expression heterogeneities of malignant cell compartments in primary SCLC. **a**, UMAP plot of malignant cells from nine SCLC patients reveals tumor-specific clusters. **b**, Violin plots showing key differentially expressed genes. **c**, Expression of *MKI67* and *TOP2A* in all malignant cells. **d**, Correlation of the proliferation score with *MKI67* expression. Correlation is represented by the Pearson correlation coefficient (r). 95% CI, 95% confidence interval. **e-g**, Heatmap of the proliferation (**e**), immune response (**f**), and hypoxia-related (**g**) programs and representative genes from the program (rows) in individual SCLC cells (columns). Cells were grouped by tumor and ordered by the single-sample GSEA (ssGSEA) score (top).

**Supplementary Fig. S4** Molecular subtype heterogeneities of SCLC. **a**, Heatmap of the z-score-normalized mean expression value of four key transcriptional regulators, namely, *ASCL1*, *NEUROD1*, *POU2F3* and *YAP1*, in each cell type. **b**, Hierarchical clustering of the relative gene expression of the four transcriptional regulators that define the subtypes of human SCLC. The color bar scale represents expression at the bulk level. The smoking status, relapse status, characteristics and subtypes are illustrated. **c** Expression of *ASCL1*, *NEUROD1* and *POU2F3* in individual cells from 19 patients in JM Chan’s study. **d**, Three branched trajectories of malignant SCLC cells in a two-dimensional state space inferred by Monocle 2. **e**, Expression levels of *ASCL1*, *NEUROD1*, *POU2F3* and *YAP1* in three trajectory branches in figure 4**c**. **f**, Pie plot illustrates the percentages of cells from each sample in the three states.

**Supplementary Fig. S5** Heterogeneous expression patterns of key TFs in SCLC. **a**, Hierarchical clustering of *ASCL1* and *NEUROD1* expression in malignant cells from samples in JM Chan’s study, including RU1231, RU1152, and RU1080. **b**-**e**, Scatter dot plot illustrates the expression of *MYC* (**b**), *CD44* (**c**), *HES1* (**d**), and *DLK1* (**e**) in the SCLC-A, SCLC-A/N, and SCLC-non-NE subtypes. Each bar represents the mean ± SD. Statistical analyses were performed using one-way ANOVA followed by Tukey’s multiple comparisons test. ***, *p*-value < 0.001; ****, *p*-value < 0.0001; ns, not significant. **f, g** SCLC cell lines, H1048 (**f**) and H69 (**g**), were incubated with control medium (PBS), silibinin, etoposide and cisplatin (EP), EP plus silibinin for 24h. Cell viability was assessed using a CCK-8 assay and values were normalized to the mean level of control samples. Each bar represents the mean ± SD of three replicates. One-way ANOVA followed by Bonferroni’s post-hoc comparisons tests were performed in all statistical analyses. *, p < 0.01, **, p < 0.001, ***, p < 0.0001. **h** Mice bearing H1048 SCLC tumors were treated with different reagents (PBS, silibinin, EP, EP plus silibinin) every 6 days for a total of 3 injections (arrows). Statistical analyses were performed using a KruskaleWallis test followed by the Manne Whitney test. *, p < 0.01, **, p < 0.001, ***, p < 0.0001.

**Supplementary Fig. S6** Functional associations of intratumor subtype signatures. **a**-**c**, GSEA plots for the hallmarks by comparing SCLC-NE vs. SCLC-non-NE (**a**), SCLC-A/N vs. SCLC-A (**b**), and SCLC-non-NE vs. SCLC-NE (**c**). **d-f**, Scores of the three subtypes regarding the cell cycle- (**d**), immune- (**e**) and hypoxia-related (**f**) hallmarks. Statistical analyses were performed using one-way ANOVA followed by Bonferroni’s multiple comparisons test, with the *p*-values indicated. **g**, Typical IHC images of ASCL1-neg, ASCL1-pos, NEUROD1-neg, and NEUROD1-pos. **h**, Overall survival (OS) analysis of SCLC patients between those with either a heterogeneous or homogeneous expression of *ASCL1* and *NEUROD1*. The log-rank test was performed to indicate a difference with a *p*-value of less than 0.05. **i**, Bar plot illustrates the numbers of putative receptor-ligand interactions between malignant SCLC-A, SCLC-A/N, and SCLC-non-NE cells and the indicated cell types. Interaction numbers were calculated by using CellPhoneDB based on the expression of receptors and their corresponding ligands in scRNA-seq data. ‘Ligand’ and ‘receptor’ on the ‘x’ axis indicate the expression of the ligand or receptor by malignant cells from SCLC-A, SCLC-A/N, and SCLC-non-NE. **j**, Bar plot shows the total number of interactions between SCLC-A, SCLC-A/N, and SCLC-non-NE clusters and other clusters or cell types.

**Supplementary Fig. S7** ITH of SCLC is recapitulated in relapsed tumors. **a**, UMAP plot of all cells from P2 by cell type. **b**, UMAP plot of all cells from P2 by cell of origin of the primary tumor, NAT and relapsed tumor. **c**, *POU2F3* expression in malignant cells from P2. **d**, Heatmap shows inferred CNVs in malignant cells (rows) from P2. Cells from NATs were used as references. Cells from different origins are indicated. Red: amplification; blue: deletion.

**Supplementary Fig. S8** SCLC subtypes associated with different immune microenvironments. **a** and **b**, Hierarchical clustering of relative gene expression of four key transcriptional regulators defining the subtypes of 50 SCLC cell lines (**a**) and 81 SCLC tumor samples described by George *et al*. (**b**). The color bar scale represents expression at the bulk level.

**SUPPLEMENTARY TABLES**

**Supplementary Table 1.** Clinical information of 11 SCLC patients for scRNA-seq analyses.

**Supplementary Table 2.** Clinical information of 14 SCLC patients for targeted RNA sequencing.

**Supplementary Table 3.** All DEGs and the top 100 DEGs in each patient are shown in Fig. 3b.

**Supplementary Table 4.** GSEA results for Fig. 3c.

**Supplementary Table 5.** IHC raw data and clinical information of 90 SCLC patients for IHC.

**Supplementary Table 6.** Ligand-receptor interactions deduced by CellPhoneDB for Fig. 5h-j.
