# Supplementary figures and images for: Single-cell transcriptomic profiling reveals the tumor heterogeneity of small-cell lung cancer
Source: Signal Transduct Target Ther. 2022 Oct 5;7:346. doi: 10.1038/s41392-022-01150-4 (PMC9532437; doi:10.1038/s41392-022-01150-4)

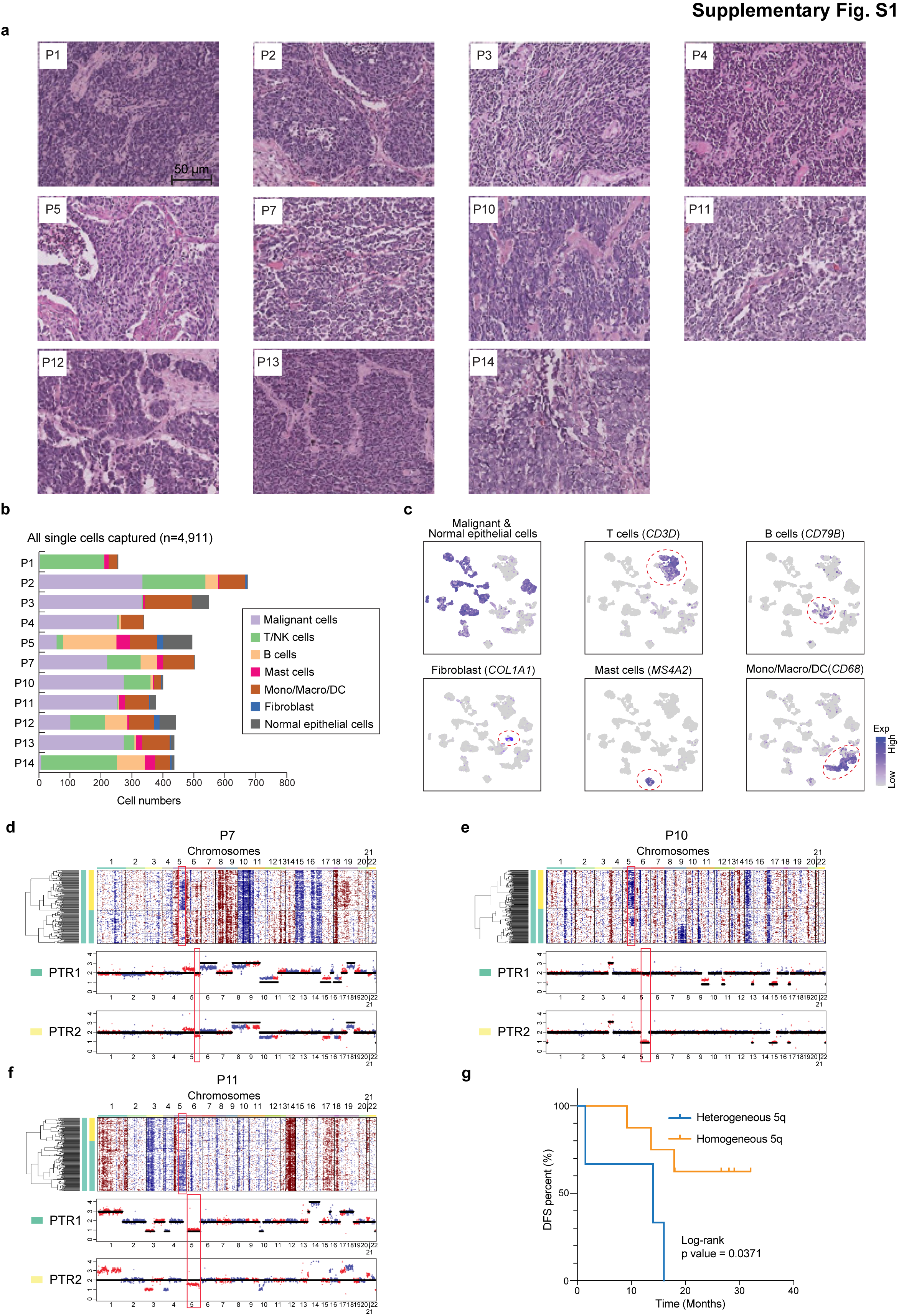

Supplement: Supplementary file 2 — Supplementary Fig. S1 [file 41392_2022_1150_MOESM2_ESM.tif]

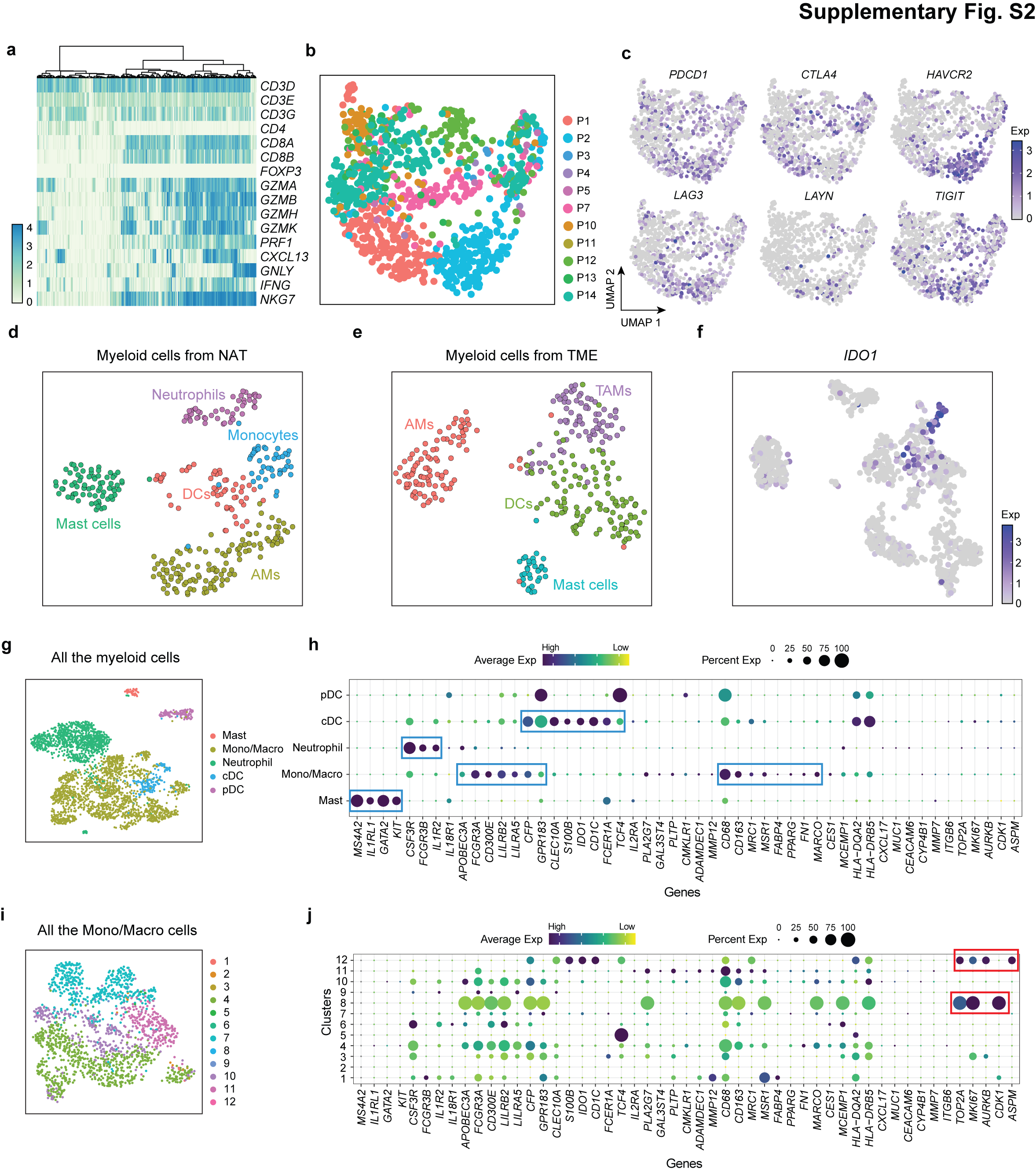

Supplement: Supplementary file 3 — Supplementary Fig. S2 [file 41392_2022_1150_MOESM3_ESM.tif]

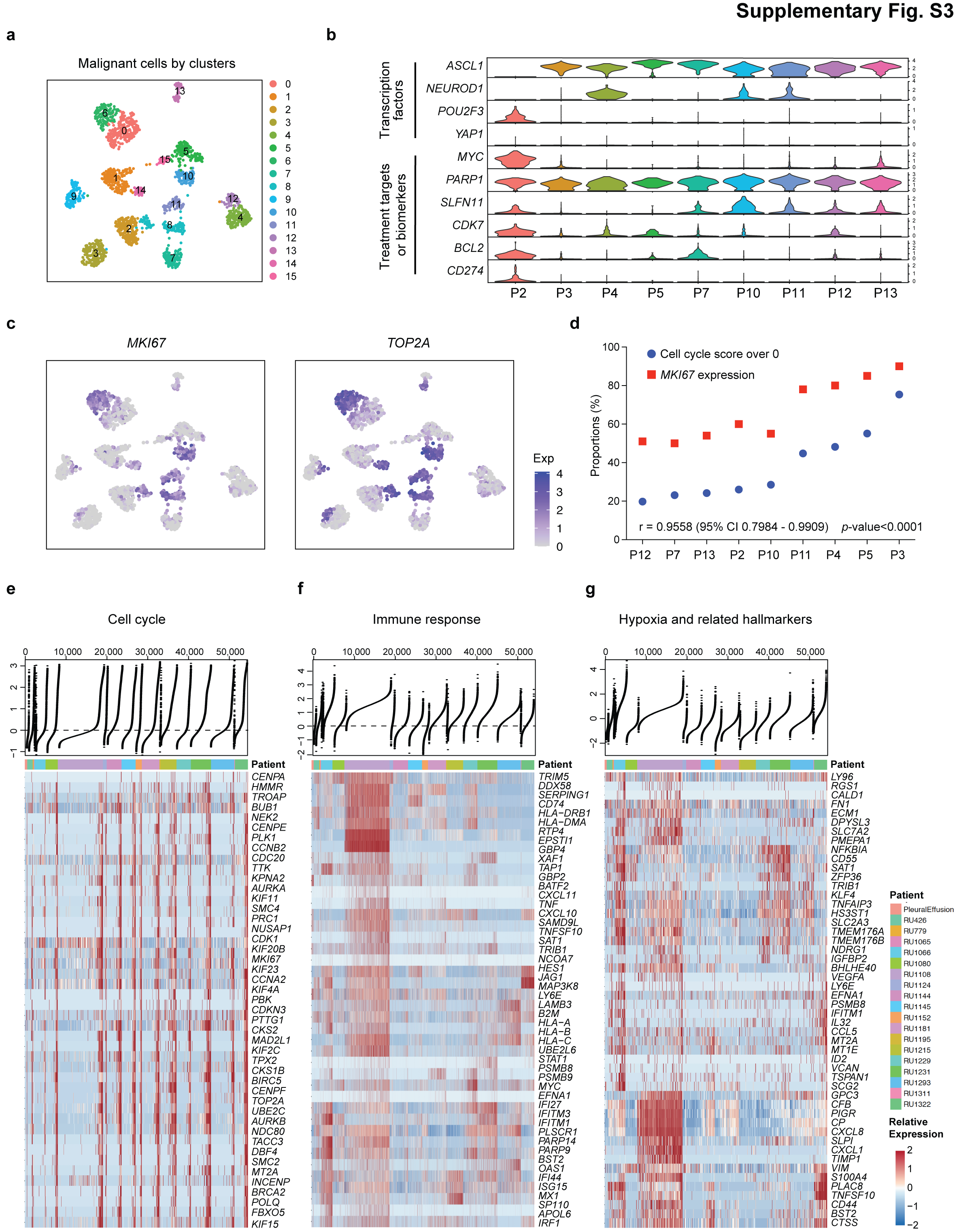

Supplement: Supplementary file 4 — Supplementary Fig. S3 [file 41392_2022_1150_MOESM4_ESM.tif]

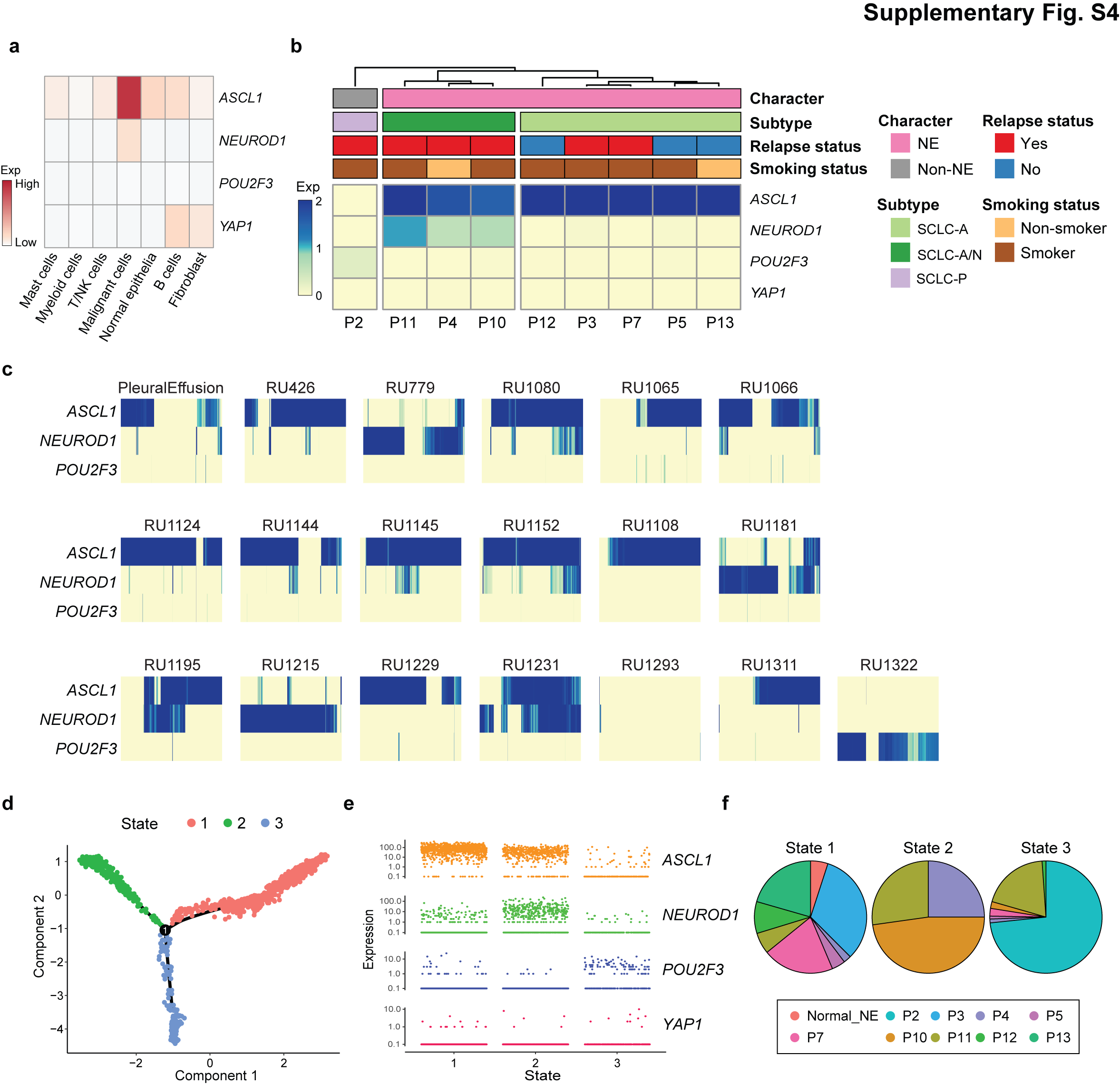

Supplement: Supplementary file 5 — Supplementary Fig. S4 [file 41392_2022_1150_MOESM5_ESM.tif]

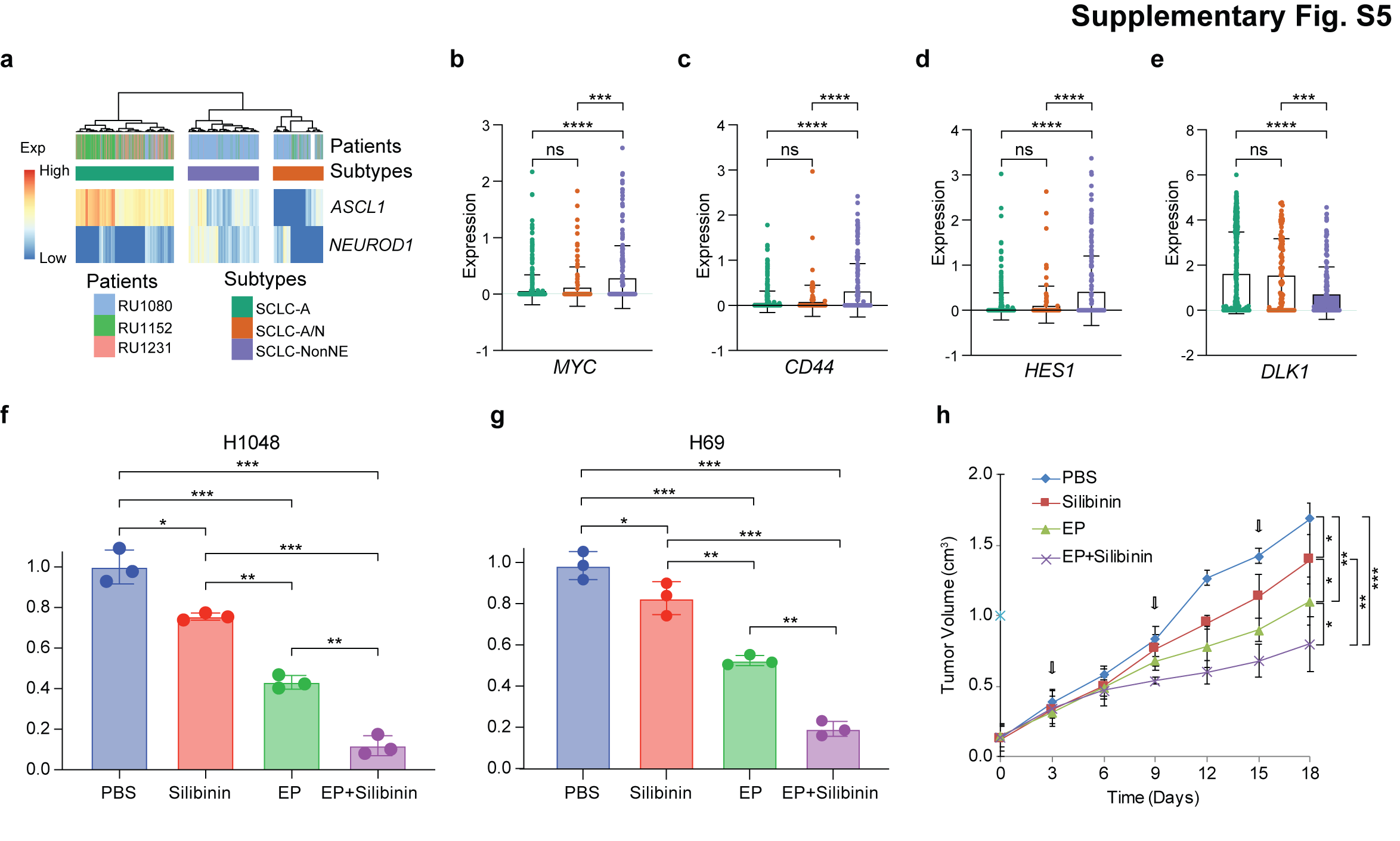

Supplement: Supplementary file 6 — Supplementary Fig. S5 [file 41392_2022_1150_MOESM6_ESM.tif]

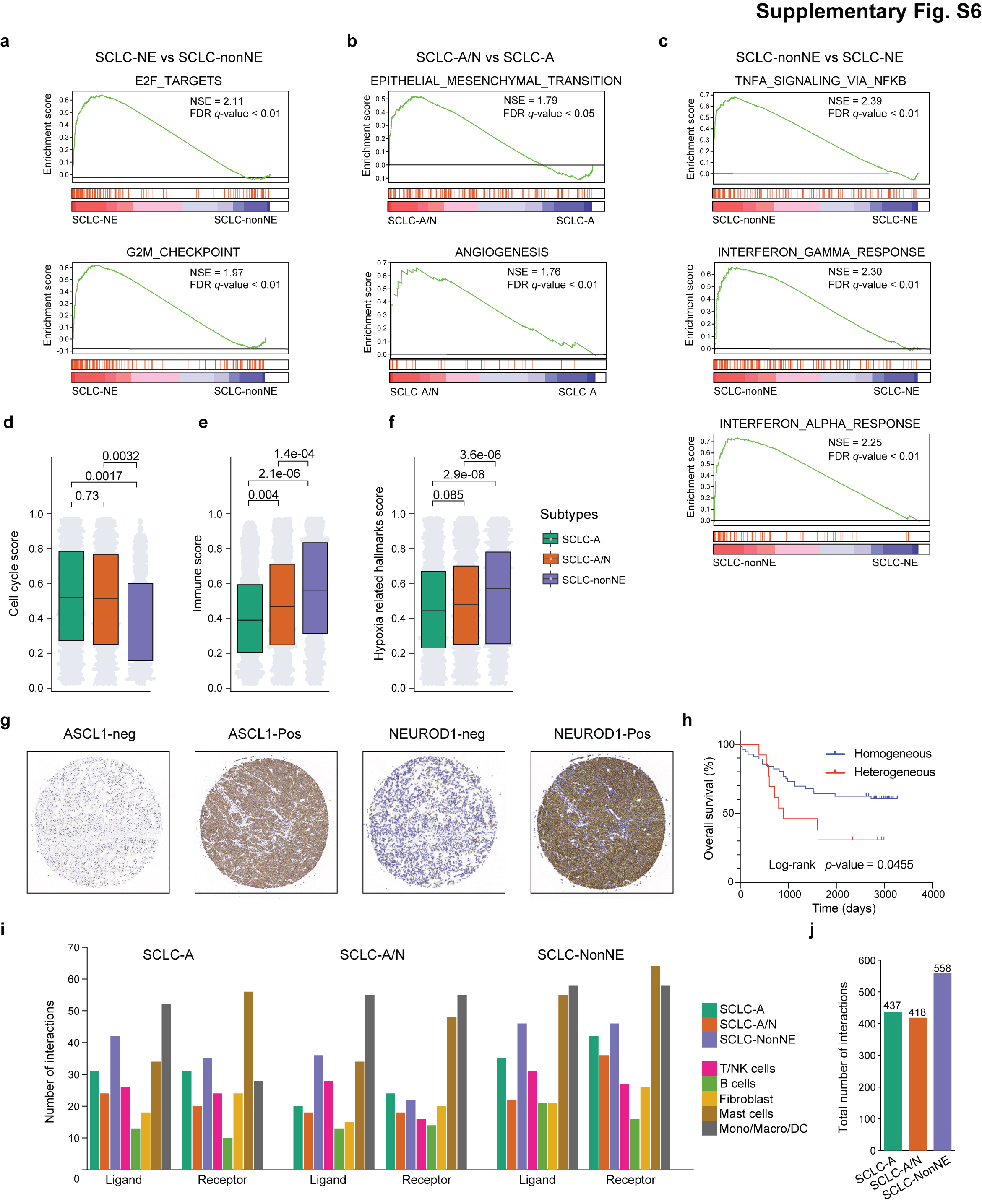

Supplement: Supplementary file 7 — Supplementary Fig. S6 [file 41392_2022_1150_MOESM7_ESM.tif]

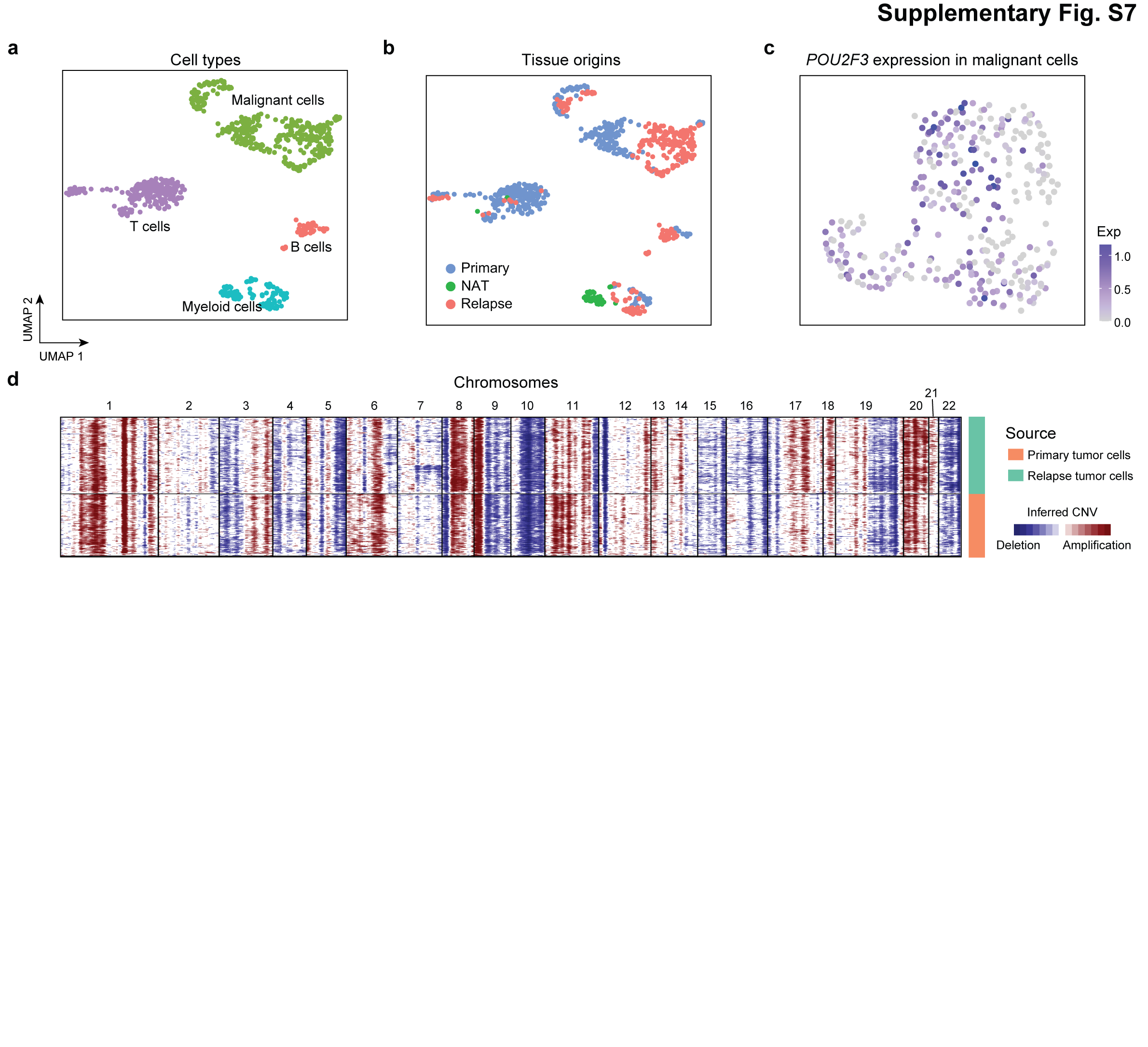

Supplement: Supplementary file 8 — Supplementary Fig. S7 [file 41392_2022_1150_MOESM8_ESM.tif]

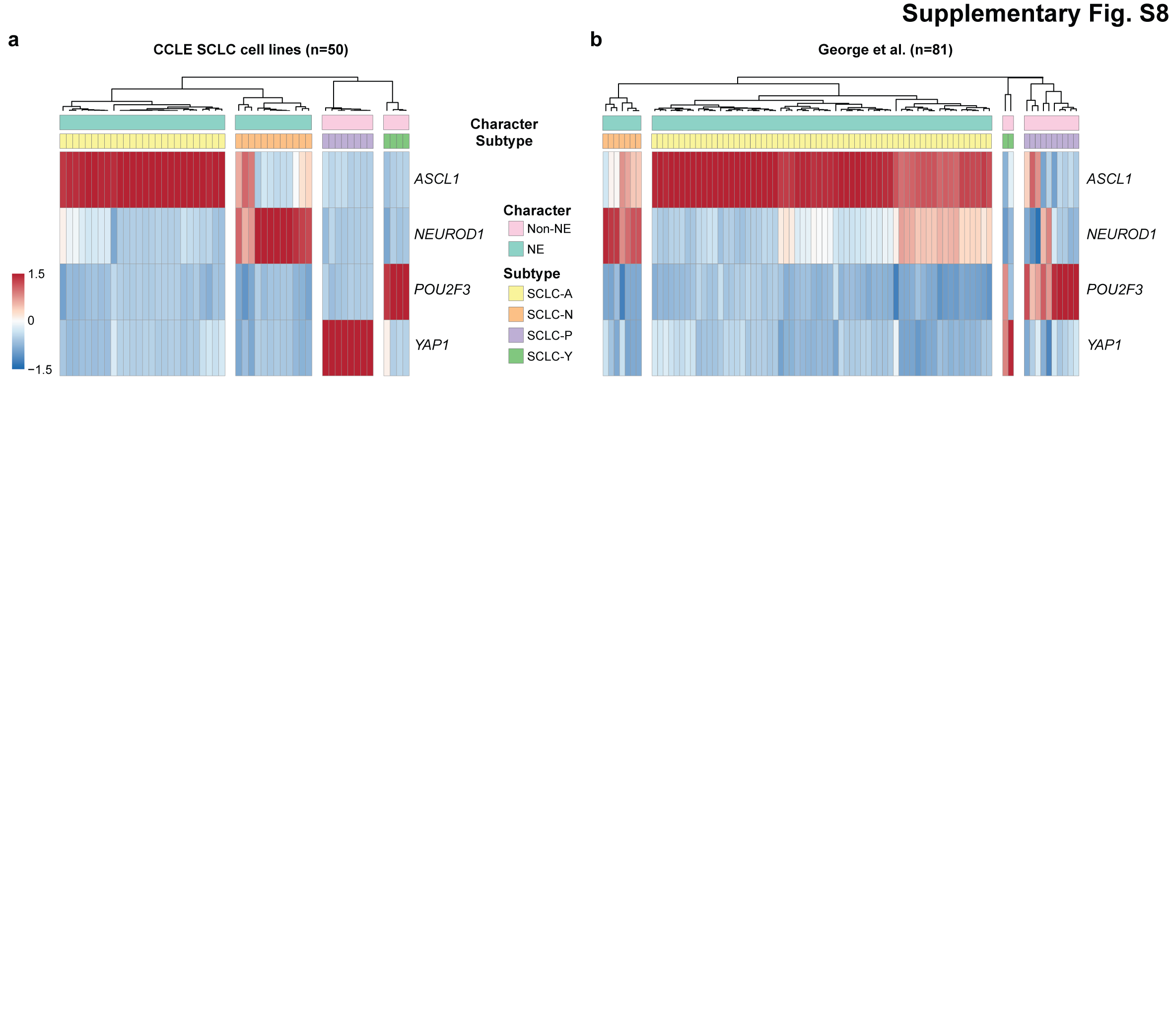

Supplement: Supplementary file 9 — Supplementary Fig. S8 [file 41392_2022_1150_MOESM9_ESM.tif]
